# Multi‐Channel Neural Interface for Neural Recording and Neuromodulation

**DOI:** 10.1002/smtd.202501227

**Published:** 2025-09-25

**Authors:** Eunmin Kim, Won Gi Chung, Enji Kim, Myoungjae Oh, Joonho Paek, Taekyeong Lee, Dayeon Kim, Seung Hyun An, Sumin Kim, Jang‐Ung Park

**Affiliations:** ^1^ Department of Materials Science and Engineering Yonsei University Seoul 03722 Republic of Korea; ^2^ Center for Nanomedicine Institute for Basic Science (IBS) Yonsei University Seoul 03722 Republic of Korea; ^3^ Department of Neurosurgery Yonsei University College of Medicine Seoul 03722 Republic of Korea; ^4^ Graduate Program of Nano Biomedical Engineering (NanoBME) Advanced Science Institute Yonsei University Seoul 03722 Republic of Korea; ^5^ Yonsei‐KIST Convergence Research Institute Seoul 03722 Republic of Korea

**Keywords:** brain‐machine interfaces, data analysis techniques, digital neurotherapies, high‐resolution recordings, multi‐channel, neural interfaces

## Abstract

Neural interfaces have emerged as pivotal platforms for advancing digital neurotherapies by enabling the real‐time acquisition and monitoring of neural signals. Traditional single‐channel systems are inherently limited in their capacity to capture the complex and large‐scale interactions among diverse neuronal populations. In contrast, multi‐channel systems provide the high spatiotemporal resolution necessary to decode the dynamic activity of neural circuits across multiple brain and spinal cord regions. This review provides a comprehensive overview of recent advances in multi‐channel neural interface technologies, encompassing both penetrating and non‐penetrating systems for high‐resolution electrophysiological recording, as well as multifunctional platforms that integrate additional modalities such as drug delivery, optical stimulation, and chemical sensing. Recent progress in this field has been driven by advances in structural and material design, including the development of soft, flexible architectures and materials for both substrates and electrodes, which improve long‐term stability and minimize tissue damage. In parallel, emerging data analysis techniques have enhanced the capacity to decode complex neural activity patterns from high‐dimensional, multi‐channel recordings. These technological advancements have broadened the potential applications of neural interfaces in brain‐machine interfaces (BMIs), facilitating precise neuromodulation, real‐time monitoring of neurological states, and integration with immersive systems such as virtual and augmented reality.

## Graphical abstract

This review highlights recent advances in multi‐channel neural interface technologies, covering both high‐resolution electrophysiological recording systems and multifunctional platforms with integrated capabilities. It also discusses innovations in structure and materials for reduced invasiveness, state‐of‐the‐art data analysis, including machine learning, and emerging applications in brain‐machine interfaces for neurotherapeutic and assistive use.

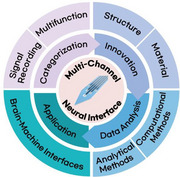

## 1 Introduction

In the field of neuroscience, the ability to record neural signals from the central nervous system, including the brain and spinal cord, is fundamentally important for elucidating the mechanisms of sensory processing, motor control, and the pathophysiology of neurological disorders. Neural interfaces facilitate the real‐time acquisition and monitoring of these signals and have recently emerged as pivotal platforms in the development of digital neurotherapies.^[^ ^1^, ^2^, ^3^, ^4^, ^5^ ^]^ The increasing prevalence of neurological and neuropsychiatric disorders, including Parkinson's disease, epilepsy, chronic pain, and spinal cord injury, underscores the urgent need for more effective and personalized treatment strategies. Digital neurotherapies, which leverage real‐time neural signal acquisition and closed‐loop neuromodulation, offer a promising avenue for non‐pharmacological, adaptive interventions. In this context, neural interfaces are not only critical tools for observing complex neural dynamics but also serve as therapeutic platforms that can interface directly with the nervous system to deliver targeted stimulation based on patient‐specific neural signatures. By addressing both monitoring and modulation needs, these technologies form the technological backbone of next‐generation clinical neurotherapies.

In conventional neural interface technology, single‐channel neural interfaces are inherently limited in their capacity to capture the complex and large‐scale interactions among diverse neuronal populations that underlie neural activity. To achieve a comprehensive understanding and effective modulation of these dynamic functional networks, multi‐channel neural interfaces with high temporal and spatial resolution have become indispensable. By enabling simultaneous recordings across multiple regions of the brain and spinal cord, these interfaces play a critical role in uncovering the spatiotemporal patterns that govern neural computation and behavior. To meet this demand, various multi‐channel neural interfaces have been developed in forms that can be implanted into or mounted on neural tissue to capture high‐resolution electrophysiological signals.^[^ ^6^, ^7^, ^8^ ^]^ With advances in materials and device engineering, these interfaces have been further enhanced to incorporate additional functionalities such as drug delivery, optical stimulation, and chemical sensing. These multifunctional platforms go beyond passive monitoring by enabling detailed assessment of disease states and supporting active modulation via stimulation or drug delivery for therapeutic and rehabilitative applications.^[^ ^9^, ^10^, ^11^ ^]^

However, traditional multi‐channel interfaces based on rigid silicon substrates face challenges in long‐term implantation due to mechanical mismatch with soft neural tissue, often leading to chronic immune responses.^[^ ^12^, ^13^ ^]^ As a result, there is growing interest in the development of minimally invasive, soft neural interfaces. Innovations in device structure—such as ultrathin, fiber, or mesh geometries—and in materials—such as polymeric, hydrogel, and liquid metal‐based substrates—have significantly improved the biocompatibility and longevity of neural interfaces.^[^ ^14^, ^15^, ^16^, ^17^ ^]^ In parallel, another major challenge is the analysis and interpretation of the vast amount of neural data collected simultaneously across multiple channels. Analytical methods for decoding connectivity between neurons in multiple regions are crucial for understanding circuit‐level function. Furthermore, advances in artificial intelligence, particularly in machine learning, are accelerating the development of more efficient and scalable tools for multi‐channel neural signal analysis.^[^ ^18^, ^19^ ^]^ These developments not only enhance our understanding of neural circuitry but also lay the foundation for advanced brain‐machine interfaces (BMIs). Multi‐channel neural interfaces serve as the foundational input layer of BMI systems, enabling the decoding of complex neural states and the implementation of closed‐loop neuromodulation strategies for therapeutic or assistive purposes.^[^ ^20^, ^21^, ^22^, ^23^, ^24^ ^]^

**Figure 1:**
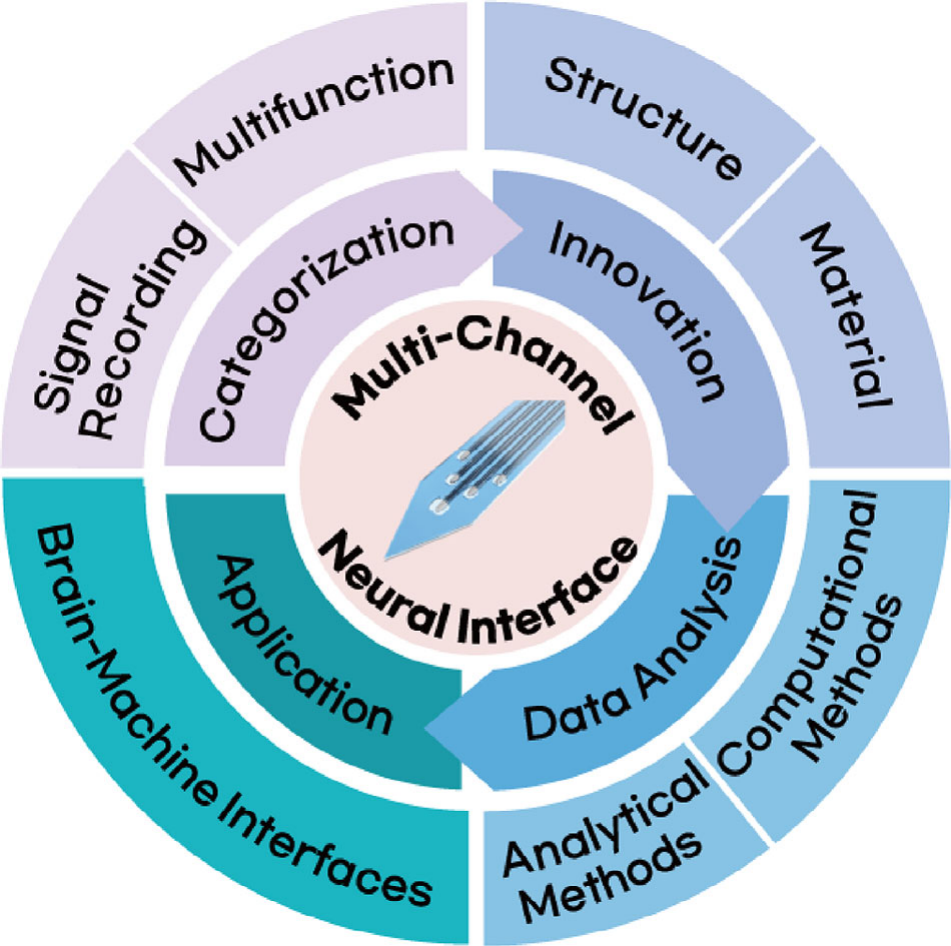
Schematic illustration of categorization, innovation, data analysis, and application of a multi‐channel neural interface.

In this review, we provide an overview of current multi‐channel neural interface technologies designed for high‐resolution electrophysiological recordings and integrated multifunctionality. We categorize these systems based on the degree of invasiveness (penetrating versus non‐penetrating) and further classify multifunctional platforms according to their integrated capabilities. We then highlight recent structural and material innovations aimed at overcoming conventional limitations, followed by a discussion of advanced neural data analysis techniques, including those leveraging machine learning. Finally, we explore the applications of multi‐channel interfaces in BMIs, outlining how these technologies are contributing to the next generation of neural decoding and neuroengineering platforms (**Figure** **1**).

## 2 Multi‐Channel Neural Interface

Recent progress in neural interface technologies has accelerated the development of high‐resolution, multi‐channel systems capable of recording distributed brain activity with fine spatiotemporal resolution. Early neural interfaces, such as the silicon‐based Michigan probe and Utah array, provided the first scalable approaches for intracortical recordings, offering modest channel counts but establishing the feasibility of multi‐site measurements.^[^ ^25^, ^26^, ^27^ ^]^ Their rigid silicon substrates enabled precise microfabrication yet posed long‐term challenges in tissue compatibility. To overcome these limitations, polymer‐based flexible arrays employing materials such as polyimide, SU‐8, and parylene‐C and mesh electrodes were introduced, yielding conformable interfaces with improved biocompatibility.^[^ ^28^, ^29^, ^30^, ^31^, ^32^, ^33^, ^34^, ^35^ ^]^ In parallel, engineering advances along the rigid‐silicon path produced Neuropixels probes, which achieved unprecedented integration densities—thousands of recording sites on a single shank—while maintaining high signal fidelity.^36,37^ More recently, soft‐material electrodes based on hydrogels and fluorinated elastomers have emerged as the next generation of ultra‐compliant neural interfaces.^[^ ^38^, ^39^ ^]^ **Table** **1** summarizes representative examples of multi‐channel neural interfaces. In addition, multifunctional platforms integrating electrical, optical, and chemical modalities have emerged to simultaneously probe and modulate neural activity. In this section, we categorize recent multi‐channel neural interfaces into high‐resolution recording systems and multifunctional systems.

**Table 1:** Technological evolution of multi‐channel neural interfaces.

Period/Year	Multi‐channel device	Electrode material	Substrate material	Refs.
1980s	Michigan probe	Au	Silicon	[25, 26]
1990s	Utah array	Pt	Silicon	[27]
2000s ∼ 2010s	Polymer based flexible electrode array	PEDOT:PSS, Ti/Pt	Polyimide	[28, 29]
		Au, Pt	SU‐8	[30, 31]
2015	NeuroGrid	PEDOT:PSS	Parylene C	[32]
2015∼	Mesh electrode	Pt	SU‐8	[33, 34, 35, 40]
2017∼	Neuropixel	TiN	Silicon	[36, 37]
2020s	Soft electrode	Pt black	PFPE‐DMA	[38]
		Sn	Hydrogel matrix	[39]

### 2.1 High‐Resolution Electrophysiological Recording

Electrophysiological signals provide a direct window into the electrical activity of neurons, enabling precise analysis of single‐cell or population‐level dynamics. Multi‐channel neural interfaces have become increasingly important in neuroscience, as they allow for the acquisition of dense and high‐quality neural data, which is critical for understanding the functional organization of neural circuits and for applications in both basic and translational research.^[^ ^8^, ^41^, ^42^, ^43^, ^44^, ^45^, ^46^ ^]^ Neural interfaces can generally be classified by their level of invasiveness. Non‐invasive systems, such as electroencephalography (EEG), are placed on the scalp and record brain signals without penetrating the body, offering safety and convenience but limited spatial resolution.^[^ ^47^, ^48^ ^]^ In contrast, invasive neural interfaces offer higher spatial and temporal resolution by interfacing directly with neural tissue, and can be divided into penetrating and non‐penetrating types depending on whether they are inserted into the tissue or placed on surface.^[^ ^7^, ^49^ ^]^ While non‐invasive systems remain valuable in many clinical and research settings, this section focuses specifically on invasive multi‐channel neural interface, which are essential for capturing fine‐scale electrophysiological signals with high fidelity.^[^ ^50^, ^51^, ^52^, ^53^, ^54^ ^]^

#### 2.1.1 Penetrating Type

Penetrating multi‐channel probes are directly inserted into the brain or spinal cord tissue, enabling high‐resolution recording of neural activity from within the neural parenchyma. These devices have been extensively used to record neuronal activity in both central nervous system structures. In the brain, they help investigate neurological disorders such as Parkinson's disease, epilepsy, and depression, and support the development of targeted therapeutic interventions.^[^ ^55^, ^56^, ^57^ ^]^ In the spinal cord, they facilitate the recording of motor and sensory activity, aiding studies on motor control, pain, and spinal cord injury recovery.

**Figure 2:**
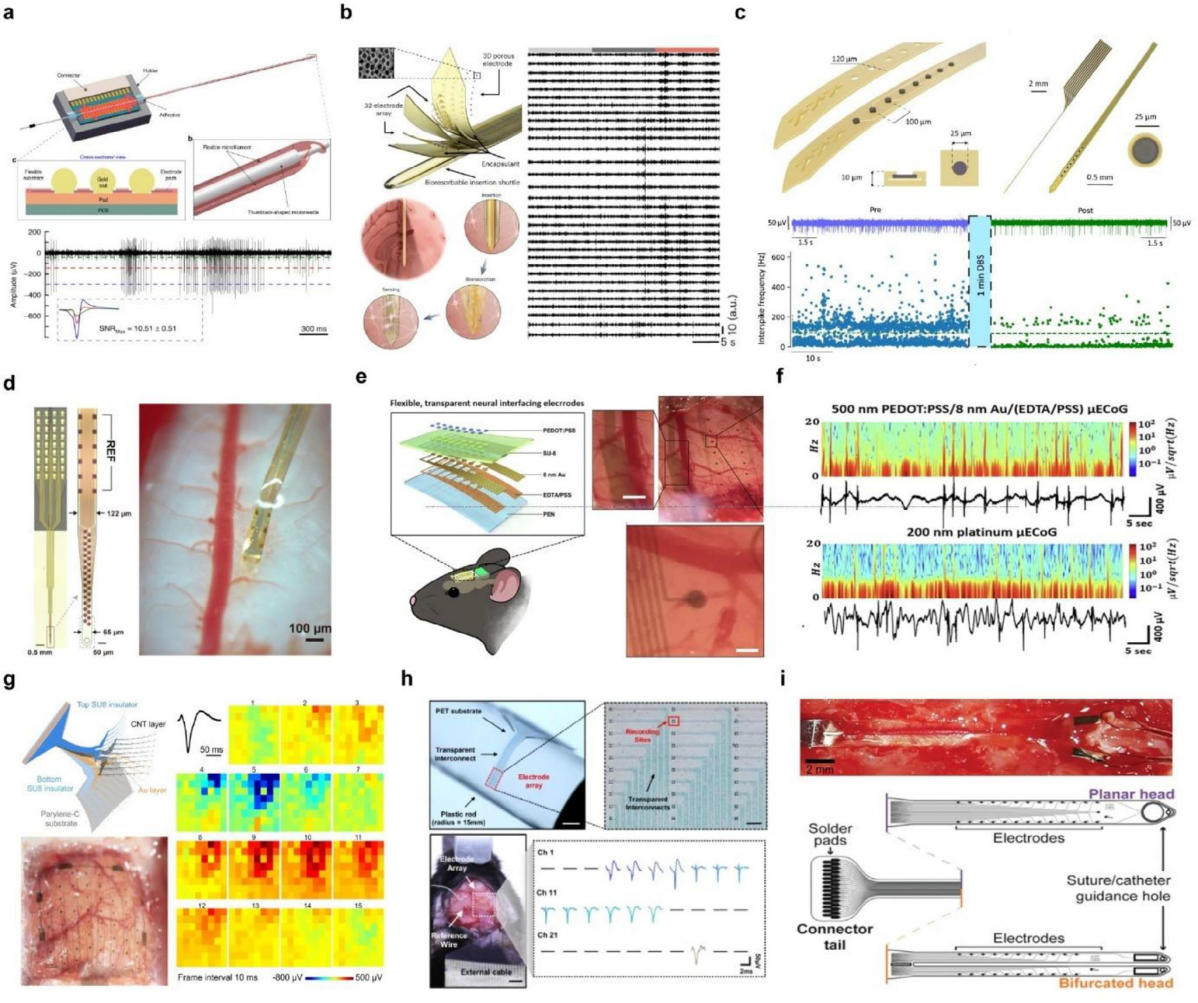
Multi‐channel probes for high‐resolution electrophysiological recording. a) Schematic illustration of fMEA (top) and neural signals acquired from fMEA recorded in PMd after 300–3000 Hz bandpass filtering (bottom). The left‐bottom panel shows the waveforms of three units after spike sorting. Reproduced with permission.^[^ ^58^ ^]^ Copyright 2023, Springer Nature. b) Schematic illustration of a flexible neural probe with 3D nanoporous microelectrodes coated with a bioresorbable insertion shuttle and the process where the shuttle guides the neural probe and dissolves to expose electrodes (left) and representative filtered LFP traces (35–50 Hz) from 32 electrodes in the LHA (right). Reproduced with permission.^[^ ^59^ ^]^ Copyright 2024, Springer Nature. c) Illustration of a microelectrode linear array with cross‐sectional image of the device layers (top) and recorded MUA spike activity in STN region of a Parkinsonian rat during pre (blue trace) and post (green trace) stimulation (bottom). Reproduced with permission.^[^ ^55^ ^]^ Copyright 2025, Springer Nature. d) Image of a 32‐channel NET showing the layout of recording and reference contacts (left) and implanted in the spinal cord (right). Reproduced with permission.^[^ ^60^ ^]^ Copyright 2024, Elsevier. e) Overall structures of ultra‐thin, transparent 16‐channel neural electrodes and optical images of transparent 500 nm PEDOT:PSS/8 nm Au/(EDTA/PSS) µECoG array (scale bar: 100 µm). f) Time‐domain analysis of the short‐time Fourier transform for each µECoG array recording. Reproduced with permission.^[^ ^61^ ^]^ Copyright 2025, Springer Nature. g) Schematic view of the layered structure of the SCEAs and an optical image showing SCEA implanted epidurally into a rat brain (left, scale bar: 1 mm). Movie frames show varied spatial‐temporal micro‐ECoG voltage patterns during a representative SWD period (right). Reproduced with permission.^[^ ^62^ ^]^ Copyright 2024, Springer Nature. h) Image of the FPE‐PEDOT on a plastic rod and a close‐up image of the 30 channel electrode arrays with 20 × 20 *µm* ^2^ recording sites (top, scale bar: 2 mm, 100 µm) and a photograph of the electrodes implanted in the somatosensory cortex and localized negative peaks in extracellular action potentials under normal conditions (bottom, scale bar: 2 mm). Reproduced with permission.^[^ ^63^ ^]^ Copyright 2025, Springer Nature. i) The bioelectronic implant consisted of a connector tail with two‐head design (bottom), and each arm was inserted into the subdural space from the left side of the image, and the tips of each arm exit the subdural space on the right side of the image (top). Reproduced with permission.^[^ ^64^ ^]^ Copyright 2022, John Wiley and Sons.

Wang et al. developed a flexible multi‐channel probe to perform extracellular recordings in the brains of non‐human primates. A guide tube was used to minimize dura damage, through which a flexible electrode array was inserted into the cortex. The flexible multielectrode array (fMEA) consisted of two parallel microfilaments (each 60 mm long), with 16 electrode sites (15 µm diameter) arranged over a 3 mm length at the tips (**Figure** **2**a).^[^ ^58^ ^]^ Micro‐slots at the tip allowed rigid microneedles to insert the flexible probes into brain tissue. Gold balls were used in place of conventional wires for signal transmission, and each electrode pad featured three vias spaced at 150 µm. Their innovative “needle‐filament‐tube” approach enabled the direct insertion of flexible electrodes without dura resection. Recordings from the premotor cortex of monkeys showed filtered signals (300–3000 Hz), and spike sorting revealed three distinct neuronal units with signal‐to‐noise ratios (mean ± SD) of 10.51 ± 0.51, 5.18 ± 1.66, and 1.83 ± 0.35. The fMEA demonstrated potential for deep brain recordings in large animals, with adjustable channel count and flexible spatial deployment. While this study focused on acute recordings, future work will require customized insertion devices for chronic applications by removing the guide tube and microneedle.

More recently, Oh et al. developed a flexible 32‐channel neural probe capable of recording neurobehavioral signals for over one month in freely moving monkeys. This battery‐free device operates via coil‐based wireless power transfer and features a bioresorbable insertion shuttle, allowing for minimally invasive implantation beneath the scalp of awake NHPs. The system consists of a ≈7.7 cm flexible neural probe and a main embedded circuit, with a structure composed of a Cr/Au bilayer electrode array and 3D nanoporous microelectrodes made of Pt/IrOx, encapsulated between thin polyimide film layers. A Litz coil receives wireless power at a center frequency of 13.56 MHz, and a 0.1 mm‐thick flexible ferrite sheet enhances magnetic induction to maximize power transfer efficiency. The sucrose‐based biodegradable shuttle enables accurate insertion of the flexible probe into deep brain regions while minimizing tissue damage during implantation. In this study, the probe was implanted in the lateral hypothalamus area (LHA) to analyze neuronal activity associated with feeding behavior. After implantation, local field potentials (LFPs) were recorded from 32 channels across 35 eating episodes. In each 36‐s trial, LFPs were segmented into three phases—craving, seeking, and consumption—with 12‐s intervals. Significant differences in gamma power were observed across these behavioral phases. This device enables unobtrusive neural recording in naturally behaving primates, offering a powerful platform for the study of innate behavior and providing a potential translational tool for investigating human brain disorders (Figure 2b).^[^ ^59^ ^]^

To enable high‐resolution, bidirectional interfacing with deep brain structures, Ria et al. developed a flexible neural probe integrating reduced graphene oxide (rGO) microelectrodes for simultaneous recording and stimulation in Parkinsonian rodents.^[^ ^55^ ^]^ The device consists of a 10 µm‐thick, 120 µm‐wide thin‐film shank incorporating eight microelectrodes (25 µm in diameter) spaced at 100 µm intervals (Figure 2c top). The rGO electrodes, characterized by a nanoporous structure with high charge injection capacity (2.3 mCcm^−^^2^) and low impedance 29.4 ± 5kΩ at 1 kHz), were patterned using CMOS‐compatible processes and encapsulated within a polyimide substrate. To facilitate deep‐brain implantation, the flexible probe was temporarily adhered to a rigid silicon shuttle via a dissolvable polyvinyl alcohol (PVA) adhesive, enabling precise targeting of the subthalamic nucleus (STN) in rats with minimal tissue damage. In vivo experiments demonstrated that the rGO microelectrodes could reliably record both LFP and multi‐unit activity (MUA) with high signal‐to‐noise ratios. During acute and chronic recordings in healthy and hemiparkinsonian rats, spatially resolved spike activity enabled functional localization of the STN‐based on differential firing patterns across the electrode array. In Parkinsonian rats induced via 6‐OHDA lesions, neurons in the STN exhibited increased bursting activity compared to control animals, serving as a potential electrophysiological biomarker (Figure 2c bottom). Moreover, delivery of high‐frequency electrical stimulation (130 Hz, 75 µA) through the same microelectrode array modulated the pathological burst activity without affecting tonic firing or LFP signatures, demonstrating focal neuromodulation within a ≈200 µm radius. These results underscore the utility of bidirectional rGO microelectrodes for closed‐loop deep brain stimulation paradigms in preclinical models of neurological disease. Although implantable multi‐channel probes allow direct access to neural circuits in the brain, applying these techniques to the spinal cord during behavior demonstrates substantial challenges due to the spinal cord's significant motion relative to the vertebrae. This has limited our understanding of how spinal neurons encode motor commands. Wu et al. addressed this by developing ultra‐flexible 1 µm‐thick polyimide NETs (nano electronic threads) capable of laminar recordings from multiple neural units in the lumbar spinal cord of freely moving mice. The extreme thinness of the polyimide electrodes reduced strain energy density at the probe–tissue interface to levels similar to that of native tissue deformation. Compared to rigid silicon electrodes, chronic implantation of these electrodes for 28 days caused minimal disruption to surrounding tissue, demonstrating their mechanical compatibility with highly mobile spinal environments. The 32‐channels NET, designed to span the 600 µm depth of the ventral spinal cord, featured a tapered geometry to reduce insertion‐related damage (Figure 2d).^[^ ^60^ ^]^ The NET was attached to a 50 µm microneedle via a “microneedle‐thread” strategy, allowing slow and precise insertion. The probe was implanted 50–250 µm lateral to the central vein and ≈1000 µm deep, targeting the ventral gray matter. Post‐insertion, the needle was removed, leaving the thread embedded in tissue. The recorded extracellular action potentials (EAPs) had a high signal‐to‐noise ratio (SNR) and distinct spike clusters, revealing diverse firing patterns during locomotion. Chronic recordings allowed reliable tracking of individual neuronal units and their functional dynamics over several days. This method presents a significant advance in understanding how spinal circuits generate and control movement.

#### 2.1.2 Non‐Penetrating Type

Non‐penetrating multi‐channel probes do not require insertion through biological tissue, thereby avoiding surgical risks and minimizing disruption to neural function. These characteristics make non‐penetrating multi‐channel probes a key technology in the diagnosis, monitoring, and therapeutic research of various brain and spinal cord disorders. In the brain, they are commonly utilized in electrocorticography (ECoG) to measure neural signals from the cortical surface. In the spinal cord, they are used to record neural activity from the surface to analyze motor function and movement‐related signals.

In the brain, non‐penetrating multi‐channel probes are primarily attached to the cerebral cortex in the form of ECoG arrays. When combined with optogenetics, they enable not only precise modulation and analysis of neural circuits but also allow for simultaneous electrical signal recording and optical imaging in live animal models. Kim et al. developed an ultra‐thin, transparent gold (Au) microelectrode array using a hexadentate metal–polymer ligand seeded on an EDTA/PSS substrate. This enabled the fabrication of a flexible and optically transparent µECoG electrode array. An ultra‐thin gold film (8 nm) was deposited on a 25 µm‐thick flexible substrate through the formation of EDTA–metal complexes. The resulting Au‐based bioelectronic interface exhibited excellent electrochemical performance (0.73 Ω·cm^2^), enabling long‐term neural recording and stimulation. In optogenetic experiments with ChR2‐expressing mice, the transparent electrodes outperformed conventional opaque electrodes by enabling artifact‐free optical stimulation and more efficient neural modulation. When placed over the parietal cortex, the transparent µECoG array allowed simultaneous optical imaging of sub‐surface vasculature and electrophysiological recording of cortical activity (Figure 2e). Power spectral density (PSD) analysis of in vivo recordings revealed spontaneous peak‐to‐peak signals in the range of 400–800 µV over 60 seconds, with a high SNR of ≈43.28 dB. Both transparent and opaque electrodes captured clear low‐frequency oscillations (<6 Hz) and spontaneous spike activity (Figure 2f).^[^ ^61^ ^]^ These findings demonstrate the utility of the ultrathin Au electrode array for transparent, minimally invasive neural interfacing and optogenetic applications.^[^ ^65^, ^66^, ^67^, ^68^, ^69^, ^70^, ^71^ ^]^

Wei et al. developed an ultrathin, flexible shape‐changing electrode array (SCEA) that minimizes surgical risks during implantation. The SCEA can be inserted in a compressed state through small openings in the skull or dura and fully deployed on the cortical surface to cover a broad area. To enhance stretchability, a carbon nanotube (CNT) thin film was added on top of the gold layer at the interconnect regions. The electrode array was encapsulated between SU8 insulating layers, with the recording sites exposed. The device was implanted using a shape actuator through a small cranial opening measuring 0.8‐2 mm (width‐length). After sufficient rinsing with saline, the actuator was carefully retracted, leaving the SCEA in place. Once implanted, the device enabled high‐resolution recordings of cortical activity. In this study, neuronal activity was recorded following pharmacologically induced seizures. The 64‐channel SCEA recorded seizure activity for ≈70 min, revealing higher‐amplitude epileptic spikes in channels near the drug injection site. Voltage maps from all channels demonstrated the spatiotemporal propagation of neural waves from the injection site to distal cortical regions. This work demonstrates that high‐quality cortical signals can be reliably recorded from both epidural and subdural spaces, enabling the measurement of physiological and pathological cortical activities with high spatiotemporal resolution, wide bandwidth, and excellent signal quality (Figure 2g).^[^ ^62^ ^]^

To achieve high‐resolution neural signal acquisition, low noise levels, device sensitivity, and biocompatibility are essential requirements for multi‐channel probes. Kim et al. developed a fully transparent, metal‐free neural electrode array, in which both the electrodes and interconnects are transparent, enabling precise recordings optimized for high‐resolution signal acquisition. The material was enhanced using formamide, phosphoric acid, and ethylene glycol, resulting in superior electrical properties. The 30‐channels array was composed of 20 × 20 µm^2^ recording sites with exceptional flexibility, capable of bending around a plastic rod with a curvature radius of 15 mm. The electrodes demonstrated a low impedance of 45.8 kΩ at 1 kHz. These PEDOT:PSS‐based transparent electrodes outperformed other metal‐free devices in terms of signal clarity and electrochemical stability, enabling precise recording of both extracellular action potentials (EAPs) and local field potentials. In vivo implantation into the primary somatosensory cortex (S1) of mice showed clear EAPs across the entire electrode array, with stable and regular firing patterns observed under baseline conditions. Compared to other electrode types, the FPE‐PEDOT array clearly captured seizure spikes with minimal noise. Its high optical transparency, superior electrochemical performance, and compatibility with brain tissue make it suitable for large‐area cortical recordings, seizure detection, and high‐resolution EAP monitoring (Figure 2h).^[^ ^63^ ^]^

A non‐penetrating multi‐channel probe for the spinal cord should adhere fully to the surface of the spinal cord and maintain structural stability even during spinal movements. In addition, it must exhibit excellent biocompatibility to ensure that it does not interfere with various motor functions associated with the spinal cord. Harland et al. developed an ultra‐thin and flexible bioelectronic implant for direct subdural contact with the thoracic spinal cord in rats. Fabricated from 8 µm‐thick polyimide, the device significantly reduced pressure on the spinal surface compared to traditional thick implants. To address the need for long‐term spinal recording and stimulation, the study compared planar and bifurcated designs, evaluating their impact on behavior and spinal morphology. As shown in Figure 2i, laminectomy was performed to expose the dorsal surface of the spinal cord, and the implant was inserted in the subdural. The bifurcated design reduces pressure on the central vein, ensuring mechanical safety and long‐term stability. The implanted device did not impair hindlimb function or alter spinal structure and remained functional for over 12 weeks in freely moving rats. Neural activity was observed across all electrodes from as early as day 7 post‐implantation, including typical spike amplitudes of 1–2 mV and rare high‐amplitude spikes up to 3 mV. These findings highlight the implant's potential for long‐term monitoring of spinal cord injury and recovery, as well as for delivering localized electrical or pharmacological therapy.^[^ ^64^ ^]^

### 2.2 Integration with Multifunctional Modalities

Multi‐channel neural probes have long served as essential tools for recording electrophysiological activity across multiple neurons with high spatial and temporal resolution.^[^ ^6^, ^72^, ^73^ ^]^ Traditionally, their role has been limited to capturing neural signals, enabling the decoding of neuronal dynamics and connectivity.^[^ ^74^, ^75^, ^76^, ^77^ ^]^ However, recent advances in neuroscience have driven the evolution of these devices beyond passive signal acquisition. A new class of multifunctional multi‐channel neural probes has emerged, integrating additional capabilities such as localized drug delivery, stimulation, and chemical sensing into a single platform.^[^ ^78^, ^79^, ^80^, ^81^, ^82^, ^83^, ^84^ ^]^ These integrated functionalities allow not only the observation of neuronal activity, but also active manipulation and monitoring of the neural network. For instance, drug delivery integrated multi‐channel probes allow precise pharmacological intervention at targeted regions, optical stimulation integrated multi‐channel probes enable closed‐loop modulation of neuronal circuits, and chemical sensors integrated multi‐channel probes offer deeper insight relating to the neurochemical changes during neural events.^[^ ^84^, ^85^, ^86^, ^87^, ^88^, ^89^, ^90^ ^]^ This convergence of recording and intervention modalities represents a significant shift in the role of neural probe in understanding and treating neurological disorders from a passive neural signal observation platform to an active neuromodulation platform.

#### 2.2.1 Drug Delivery Integrated

Evaluating the neurological and behavioral effects of pharmacological agents is essential not only for the development of therapeutic interventions but also for advancing our understanding of the mechanisms underlying neurological disorders. Multi‐channel neural probes integrated with drug delivery capabilities offer a powerful platform for such investigations.^[^ ^30^, ^96^ ^]^ By enabling the localized administration of drugs and the simultaneous real‐time monitoring of neuronal activity, these multifunctional devices facilitate precise assessment of drug efficacy and neural response within targeted brain regions.

**Figure 3:**
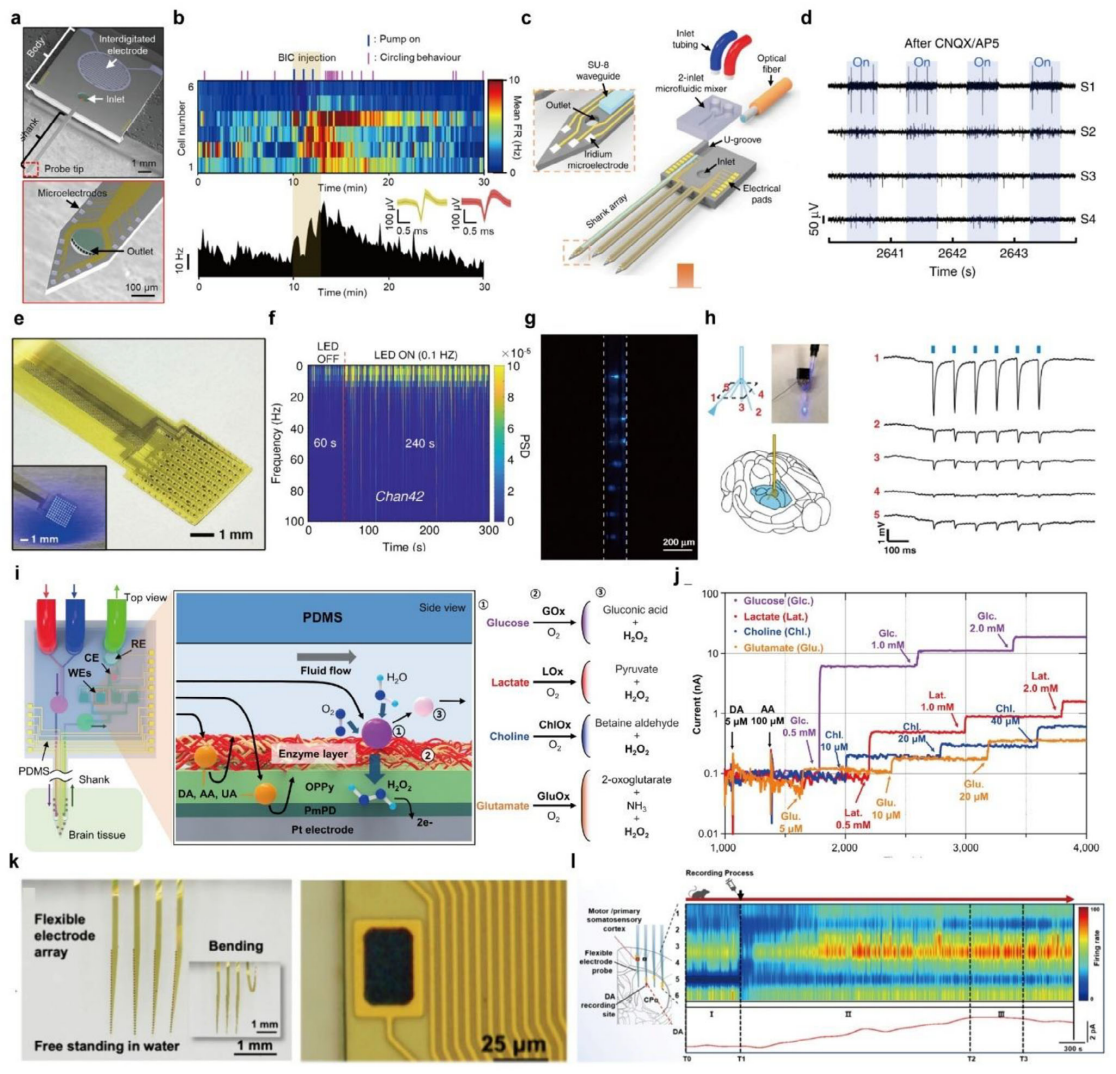
Multi‐channel neural interfaces with multifunctional modalities. a) Scanning electron microscope image of the multi‐channel neural probe with 16 microelectrodes and microfluidic channel outlets. b) Real‐time behavioral analysis, neural activities of SN neurons, and mean firing rate of SN neurons in response to bicuculine injection. Reproduced with permission.^[^ ^85^ ^]^ Copyright 2022, Springer Nature. c) Schematic illustration of multifunctional multi‐shank MEMS neural probe with 2‐inlet microfluidic mixer. d) Neural recording after CNQX and AP5 injection during optical stimulation. Reproduced with permission.^[^ ^91^ ^]^ Copyright 2019, Springer Nature. e) Optical image of microLED array‐based optogenetics device with ECOG array. f) Frequency spectrum signals recorded for 300 s, where the initial 60 s is absent from LED light stimulation and proceeding 240 s is with LED light stimulation. Reproduced with permission.^[^ ^92^ ^]^ Copyright 2024, American Chemical Society. g) Optical image of eight optical stimulation sites formed by the eight waveguide windows in the fiber probe. h) Schematic illustration of the fiber probe targeting optical stimulation to the thalamus region of the transgenic mouse brain (right). Electrophysiological recording from five recording electrodes during optical stimulation. Reproduced with permission.^[^ ^93^ ^]^ Copyright 2020, Springer Nature. i) Schematic illustration of the enzyme‐based neurochemical sensing mechanism of RTBM MEMS neural probe. j) Oxidation current responses of 4 different neurochemicals after injection of interferents. Reproduced with permission.^[^ ^94^ ^]^ Copyright 2023, National Academy of Sciences. k) Microscopic image of flexible four shank probe with 32 microelectrodes (right) and the close view of the electrode region (left). l) Firing rate changes of measured neural spikes and dopamine signal with drug injection. Reproduced with permission.^[^ ^95^ ^]^ Copyright 2024, American Chemical Society.

Yoon et al. developed a wireless, multifunctional neural probe system that integrates 16‐channel electrophysiological recording, precise drug delivery via a miniaturized electrolytic pump, and bi‐directional wireless communication, enabling chronic in vivo neuromodulation studies in freely behaving mice.^[^ ^85^ ^]^ The core of the system is a silicon‐based neural probe embedded with 16 platinum microelectrodes and integrated microfluidic channels for localized drug infusion (**Figure** **3**a). The miniaturized electrolytic pump operates by generating pneumatic pressure through electrolysis, allowing for accurate, repeatable, and dose‐controllable delivery of pharmacological agents into targeted brain regions. To ensure long‐term reliability, the device includes a refillable drug reservoir and integrated check valves that effectively prevent backflow. To support untethered behavioral experiments, the system incorporates a bi‐directional Bluetooth Low Energy (BLE) module, enabling wireless control of drug infusion and simultaneous neural signal transmission from the multi‐channel array.^[^ ^97^, ^98^ ^]^ Despite its multiple integrated components, the complete system weighs only ≈4.6 g, making it suitable for chronic implantation in mice without interfering with natural locomotor behavior, as verified through open‐field testing. The developed platform was utilized to investigate the neuromodulatory effects of GABAergic agents in vivo. Specifically, bicuculline (a GABA_A receptor antagonist) and muscimol (a GABA_A receptor agonist) were delivered into the substantia nigra (SN) and lateral hypothalamus (LH), respectively. Real‐time monitoring enabled the observation of electrophysiological and behavioral changes, such as compulsive circling and suppressed feeding behavior (Figure 3b). Furthermore, the system supported dual‐animal experiments involving food competition assays, wherein simultaneous neural recordings were obtained from both the drug‐modulated and control subjects. These results highlight the system's capacity to dissect complex neural circuit mechanisms associated with both individual and social behavior modulation.

Shin et al. developed a multifunctional neural probe system with microfluidic staggered herringbone mixer, termed µSHM, that enables simultaneous multisite electrical recording, localized drug delivery, and optical stimulation.^[^ ^91^ ^]^ The probe features four shanks, each equipped with eight iridium recording electrodes, allowing for 32‐channel electrophysiological recording. Notably, one of the shanks integrates a SU‐8‐based optical waveguide and three parallel microfluidic channels, enabling the delivery of multiple neurochemical agents with independent control (Figure 3c). The fluidic system is capable of switching drug inputs within approximately 10 s, with a swept volume of ≈40 nL, facilitating spatiotemporally precise chemical modulation of neural circuits. To demonstrate its functional capabilities, the probe was implanted into the hippocampus of mice. Optical stimulation of the CA3 region elicited synchronized neural activity in the downstream CA1 region, confirming the intact connectivity of the CA3–CA1 circuit. Subsequently, AMPA and NMDA receptor antagonists (CNQX and AP5) were infused into the CA3 via the microfluidic channels, resulting in a marked reduction of evoked responses in CA1 (Figure 3d). This pharmacological inhibition was reversible, with CA1 activity gradually recovering over time, validating the probe's ability to modulate synaptic transmission in vivo. Although the system also supports optical stimulation, the present study primarily focused on electrical recording and chemical delivery. These works demonstrate the utility of multi‐channel drug delivery‐integrated multi‐channel probes for dissecting long‐range neural circuitry by enabling simultaneous manipulation and monitoring of distinct nodes within functional brain networks.

#### 2.2.2 Optical Stimulation Integrated

Understanding the functional architecture of neural circuits and their perturbations in disease states requires tools that can manipulate and monitor neuronal activity with high precision.^[^ ^99^, ^100^ ^]^ Multi‐channel neural probes equipped with optogenetic stimulation capabilities offer a unique platform for such investigations.^[^ ^66^, ^81^, ^101^, ^102^ ^]^ By delivering light stimuli to genetically defined neuronal populations while simultaneously recording electrophysiological signals, these multifunctional probes enable cell‐type‐specific, temporally precise modulation of neural activity.^[^ ^103^, ^104^, ^105^, ^106^, ^107^ ^]^ This integrated approach facilitates the causal analysis of brain function and has proven valuable in elucidating the pathophysiology of various neurological and psychiatric disorders.

Gu et al. developed a flexible, large‐scale optogenetic interface integrating dense arrays of micro‐light‐emitting diodes (microLEDs) and electrocorticography (ECOG) electrodes for simultaneous neural stimulation and recording in vivo.^[^ ^92^ ^]^ The device is fabricated on a 12.9 µm‐thick polyimide substrate and incorporates up to 256 blue‐emitting microLEDs (110 × 110 µm^2^) and 128 ECOG electrodes arranged in a planar architecture (Figure 3e). With a pitch of 300 µm between optical and electrical units, the array enables localized optical stimulation and high‐fidelity recording across wide cortical areas. The microLEDs are thermally bonded and independently addressable via a custom controller supporting voltage modulation (1.0–4.0 V) and frequency control (0.1–500 Hz), allowing precise spatiotemporal modulation of neural circuits. Importantly, the device maintains low thermal load (<2 °C at 2.8 V), ensuring tissue safety during prolonged use. Signal artifacts induced by optical stimulation were effectively mitigated using a local average subtraction method, preserving the integrity of neural recordings during stimulation. For in vivo experiment, the device was implanted on the cortical surface of ChR2‐expressing rats, enabling simultaneous optical stimulation and ECOG recording. Upon patterned blue‐light stimulation (2.8 V, 50 ms, 0.1 Hz), the device elicited enhanced neural activity in the theta (4–8 Hz) and alpha (8–14 Hz) bands, with spectral changes localized to areas adjacent to the active microLEDs. Moreover, individually activating neighboring microLEDs resulted in distinct, spatially resolved neural responses, demonstrating the device's capacity for fine‐scale circuit interrogation and precise neuromodulation (Figure 3f).

Jiang et al. developed a multifunctional, fiber‐based neural probe using a thermal fiber drawing process that enables the co‐integration of optical, electrical, and fluidic modalities into a single flexible platform.^[^ ^93^ ^]^ The probe consists of a polycarbonate (PC) cladding layer with a central PVDF core serving as an optical waveguide (Figure 3g). During fabrication, mechanical grooves were introduced along the fiber, which were selectively filled with either 25 µm‐diameter BiSn alloy microwires for electrophysiological recording or left open to serve as microfluidic channels for chemical delivery. These components were passivated and protected by the surrounding polymer structure, ensuring mechanical stability, and biocompatibility. The fiber probes were designed to be flexible and capable of insertion over a wide spatial range via a spiral scaffold delivery mechanism, enabling broad‐area brain access with minimal tissue damage. The tip design allowed precise placement in anatomically distributed regions. In a proof‐of‐concept demonstration, the fibers were implanted in both the CA1 and CA3 regions of the mouse hippocampus. Simultaneous recordings of SU activity and LFPs were performed, along with real‐time monitoring of neural responses to optical stimulation and pharmacological delivery (Figure 3h). These works highlight the potential of ultra‐dense, optically integrated multi‐channel neural probes for mapping and manipulating functional brain circuits at the neuronal scale, and demonstrate their applicability for advanced studies in neuroscience and neuroengineering.

#### 2.2.3 Chemical Sensing Integrated

In addition to electrophysiological recordings, monitoring the local dynamics of neurotransmitters is critical for advancing our understanding of neural circuit function and the pathophysiology of brain disorders.^[^ ^108^, ^109^, ^110^, ^111^, ^112^, ^113^ ^]^ To address this issue, multi‐channel neural probes integrated with chemical sensing capabilities have been developed. These multifunctional devices enable the simultaneous detection of neurochemical signals and electrical activity, offering complementary insights into the interplay between neurotransmitter fluctuations and neuronal firing. Such integrated platforms are essential for decoding complex neuromodulatory processes in both physiological and pathological states.

Chae et al. developed a real‐time bimodal (RTBM) neural probe capable of simultaneously monitoring electrophysiological activity and neurochemical dynamics in vivo.^[^ ^94^ ^]^ The probe was fabricated on a silicon substrate using standard MEMS processes and integrates two distinct electrode arrays: 4 platinum‐based working electrodes (WEs) for neurochemical sensing and 12 platinum recording electrodes for neural signal acquisition, including LFPs and SU activity. To enable selective neurochemical detection, the WEs were individually modified with oxidase enzymes, such as glucose oxidase, lactate oxidase, glutamate oxidase, and choline oxidase, using a crosslinking method involving bovine serum albumin (BSA) and glutaraldehyde. These oxidase enzymes enable the detection of glucose, lactate, glutamate, and choline, respectively. It is important to note that choline, although closely related to acetylcholine, is a distinct excitatory neurotransmitter and was specifically targeted in this study.^[^ ^114^ ^]^ These enzyme‐coated electrodes form an electrochemical sensing array capable of detecting transient changes in extracellular concentrations of key metabolic neurotransmitters (Figure 3i). The entire probe was passivated with a parylene‐C layer, with selective openings to expose only the active sensing sites for stable operation in biological environments. For in vivo validation, the probe was implanted into the medial prefrontal cortex (mPFC) and hippocampus of mice. Local infusion of potassium chloride (KCl) was used to induce transient neural activation. The RTBM probe successfully recorded both electrophysiological signals and neurochemical fluctuations in real time, revealing region‐specific responses to the chemical perturbation (Figure 3j). The simultaneous acquisition of electrical and chemical signals demonstrated the probe's ability to characterize the interplay between neuronal activity and neurochemical signaling across distributed brain regions.

Wang et al. developed an ultraflexible neural probe that enables simultaneous wideband electrophysiological recording and electrochemical dopamine sensing over extended periods.^[^ ^95^ ^]^ The probe is fabricated on a polyimide (PI) substrate, onto which 128‐channel gold (Au) microelectrodes were patterned using standard photolithography and microfabrication techniques (Figure 3k). To enhance electrical performance and reduce impedance at the tissue interface, the electrode surfaces were electrochemically coated with reduced graphene oxide (rGO) and poly(3,4‐ethylenedioxythiophene):poly(styrenesulfonate) (PEDOT:PSS). To enable neurochemical monitoring, a subset of electrodes was modified with a Nafion thin film, forming a selective electrochemical sensor for dopamine. This sensor exhibited stable and linear detection of dopamine in the 1–100 µM concentration range, with a response time suitable for real‐time neurotransmitter monitoring in vivo. The mechanical flexibility and ultrathin profile of the probe allowed conformal attachment to the brain surface and chronic implantation with minimal tissue response. In vivo experiments were conducted in mice by implanting the probe into the primary motor cortex (M1) and caudate‐putamen (CPu). Following systemic administration of nomifensine, a dopamine reuptake inhibitor, the probe enabled concurrent measurement of LFPs, SU activity, and transient elevations in extracellular dopamine levels (Figure 3l). These synchronized multimodal recordings provided insight into the interplay between dopaminergic signaling and neural activity during pharmacological modulation. These works demonstrate the feasibility of multifunctional neural interfaces for synchronized neurochemical and electrophysiological monitoring, offering a valuable platform for studying neuromodulation and neuropharmacology in vivo.

## 3 Current Progress in Multi‐Channel Neural Interface

As multi‐channel neural interface technologies continue to advance, their role is expanding beyond simple electrophysiological signal acquisition toward more comprehensive goals—such as minimizing invasiveness, enhancing tissue integration, and enabling intelligent, closed‐loop modulation of neural circuits. This shift reflects a broader transformation in the field, where the focus is moving from passive signal collection to actively interacting with complex, distributed networks in the brain and spinal cord. Achieving such functionality requires next‐generation systems to combine structural and material innovations with robust, scalable computational tools capable of interpreting high‐dimensional neural data in real time.^[^ ^115^, ^116^, ^117^, ^118^ ^]^

This section introduces key approaches that are shaping the next generation of multi‐channel neural interfaces. We first examine structural and material strategies—such as compliant substrates and miniaturized geometries—that help reduce mechanical mismatch and invasiveness. The focus then shifts to neural data processing, where multi‐site, high‐density recordings demand sophisticated analytical techniques. Among these, machine learning has emerged as a crucial framework for identifying hidden patterns, decoding dynamic neural states, and mapping functional connectivity across large‐scale networks. These advances not only enhance our understanding of neural computation, but also form the foundation for translational applications. Accordingly, we conclude this section by presenting emerging brain‐machine interface (BMI) systems that integrate multi‐channel neural recordings and machine learning to realize closed‐loop modulation and neuroprosthetic control.

### 3.1 Structural and Material Innovations for Minimal Invasiveness

Minimizing the physical dimensions and mechanical stiffness of neural implants is critical for achieving reliable, high‐resolution electrophysiological recordings.^[^ ^16^, ^119^, ^120^, ^121^, ^122^ ^]^ Traditional devices, consisting with rigid metallic electrodes and stiff silicon substrates, often elicit strong foreign body reactions (FBRs), as the immune system perceives them as threats and responds with fibrotic encapsulation, isolating the probe from surrounding neurons and degrading recording quality. Moreover, large or rigid implants can permanently alter the local neural environment, making it difficult to capture physiological activity in its natural state. To address these challenges, recent strategies in neural interface engineering have emphasized structural and material innovations aimed at reducing invasiveness, enhancing mechanical compatibility with soft tissue, and improving long‐term biocompatibility. By closely matching the mechanical properties of brain or peripheral nerve tissue, and minimizing surgical trauma during implantation, these approaches mitigate scarring, maintain stable neuron‐probe interfaces, and enable long‐term functional integration. Such innovations represent a paradigm shift in neural interface design prioritizing chronic integration, reduced inflammatory response, and high‐fidelity signal quality through soft, flexible architectures. The characteristics of various device structures as well as different interfaces, substrates, and packaging materials for the development of these soft devices are listed in Tables 1 and **2**.

**Table 2:** Mechanical properties of different structures. *E* is the Young's modulus; *h* and *w* are the thickness and width of the device.

Structure	Thickness	Bending Stiffness			Refs.
		Definition	Unit	Value	
Mesh Structure	∼1 µm	$D=\frac{E_{S}}{12}\;\left(wh^{3}-w_{m}h_{m}^{3}\right)$	N⋅m^2^	1.26×10^−15^	[124]
	∼1 µm	$D_{m}=\frac{E_{m}b_{m}h_{m}^{3}}{12b}+\frac{E_{S}}{b}\left(\frac{\left(b-b_{m}\right){\left(2h+h_{m}\right)}^{3}}{12}+\frac{1}{6}b_{m}h^{3}+2b_{m}h^{3}+2b_{m}h{\left(\frac{h}{2}+\frac{h_{m}}{2}\right)}^{2}\right)$	N⋅m	6×10^−12^	[132]
Hydrogel Hybrid Structure	334 µm	*K_theory_* = (3π*Ed* ^4^) (64*L* ^3^)^−1^	N m^−1^	7	[39]
Thread Structure	1 µm	$\;K_{NET}=E_{SU8}\;\left(\frac{h^{3}w}{12}-\frac{h_{m}^{3}w_{m}}{12}\right)+\frac{E_{m}h_{m}^{3}w_{m}}{12}$	N⋅m^2^	10^−15^	[31]
Serpentine Structure	30 µm	${\bar{EI}}_{device}=\sum_{i=1}^{3}{\bar{E}}_{i}h_{i}\left[{\left(b-\sum_{j=1}^{i}h_{j}\right)}^{2}+\left(b-\sum_{j=1}^{i}h_{j}\right)h_{i}+\frac{1}{3}h_{i}^{2}\right]$	N⋅m	≈10^−9^	[133]

#### 3.1.1 Structural Innovations

**Figure 4:**
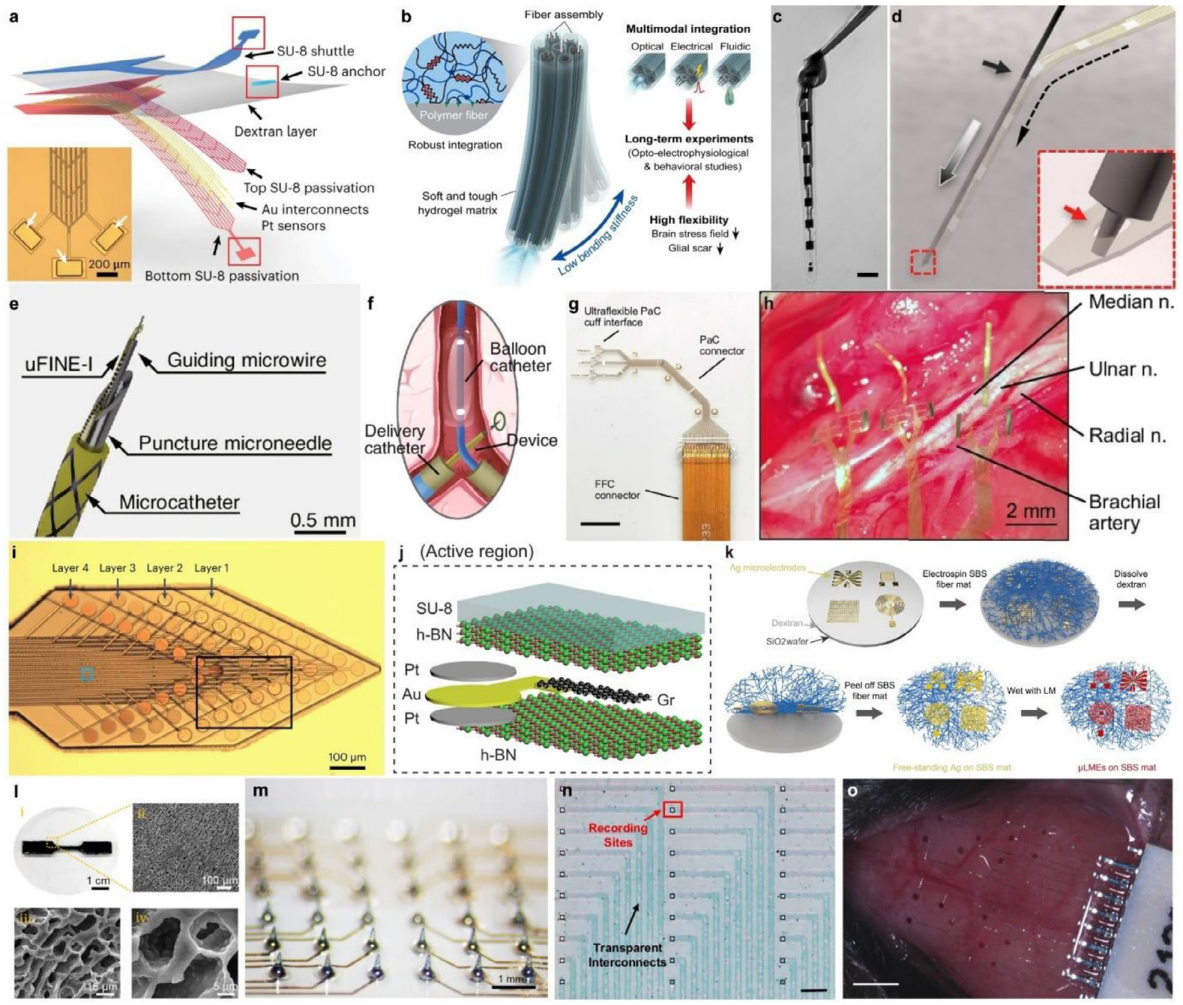
Representative structure and material innovations in neural interfaces. a) Exploded image of mesh electronics for seamless integration with neuron networks. Reproduced with permission.^[^ ^124^ ^]^ Copyright 2023, Springer Nature. b) Schematic of the multifunctional hydrogel probe for minimally invasive implantation. Reproduced with permission.^[^ ^39^ ^]^ Copyright 2021, Springer Nature. c) Image of a NET‐50 probe submerged in water. Scale bar, 50 µm. d) Schematic of the NET probe implantation process utilizing a microwire shuttle. Reproduced with permission.^[^ ^31^ ^]^ Copyright 2017, American Association for the Advancement of Science. e) Schematic illustrating the different layers that make up the device tip. f) Illustration depicting the use of a balloon catheter to carefully position the device for implantation. Reproduced with permission.^[^ ^125^ ^]^ Copyright 2024, Springer Nature. g) Image of the ultraconformable cuff implant designed for simultaneous recording and stimulation of peripheral nerves. h) Photograph of the nerve cuff implanted in the forearm of a rat. Reproduced with permission.^[^ ^126^ ^]^ Copyright 2024, Springer Nature. i) Multilayer neural probe constructed with PFPE‐DMA substrate enabling high‐density 3D electrode stacking. Reproduced with permission.^[^ ^38^ ^]^ Copyright 2023, Springer Nature. j) Schematic of NeuroWeb platform using h‐BN dielectric layers for vertical integration of multilayer electrodes. Reproduced with permission.^[^ ^127^ ^]^ Copyright 2023, Springer Nature. k) Fabrication process of µLME arrays on electrospun SBS fiber mats using photolithography‐free printing of liquid metal. Reproduced with permission.^[^ ^128^ ^]^ Copyright 2023, American Association for the Advancement of Science. l) Morphological and structural characterization of porous PEDOT:PSS‐based hydrogel microneedles. m) Fabricated hydrogel microneedle array integrated into a multi‐channel electrode platform. Reproduced with permission.^[^ ^129^ ^]^ Copyright 2024, American Association for the Advancement of Science. n) Transparent PEDOT:PSS electrode array patterned on PET for simultaneous optical imaging and electrophysiology. Scale bar, 100 µm. Reproduced with permission.^[^ ^63^ ^]^ Copyright 2025, Springer Nature. o) Fully printed liquid‐metal‐based neural interface conformally integrated on the curved skull surface. Scale bar, 1 mm. Reproduced with permission.^[^ ^49^ ^]^ Copyright 2024, American Association for the Advancement of Science.

The elastic modulus of brain tissue ranges from 0.1 to 10 kPa, and a mismatch between this soft environment and the stiffness of implanted multi‐channel devices can induce glial scarring and FBRs at the tissue–device interface.^[^ ^123^ ^]^ To mitigate this issue, mesh electronics have been extensively studied for seamless integration of neural probes within the brain's 3D tissue architecture, as they can closely match its mechanical properties near brain tissue ranges by their structural design. Zhao et al. developed a novel implantation method using a detachable polymer shuttle to deliver fully unfolded mesh electronics into the mouse brain.^[^ ^124^ ^]^ The devices were fabricated using standard photolithography, consisting of 15 µm diameter electrodes encapsulated in SU‐8, with Cr/Au interconnects (10 µm wide, <1 µm thick). **Figure** **4**a shows an exploded illustration of the mesh implant. The mesh electronics achieved a high filling ratio (73.3%) and an ultralow bending stiffness (≈10^−15^ N·m^2^), matching the mechanical properties of brain tissue. A 25‐µm‐thick polymer shuttle was temporarily attached to the mesh using a thin polyethylene glycol (PEG) layer, which could be dissolved in aqueous solution to release the probe after insertion. This approach preserved the open, macroporous structure of the mesh and enabled high‐density integration with minimal tissue disruption. Post‐implantation tissue imaging revealed an acute damage area of only 0.0068 ± 0.0017 mm^2^, indicating minimal scarring. Immunostaining at 2, 6, and 12 weeks, and 1‐year post‐implantation demonstrated that mesh electronics induced significantly less astrocyte and microglia proliferation compared to conventional thin‐film electronics, while maintaining stable neuronal density at the probe–tissue interface. Long‐term electrophysiological recordings in adult mice confirmed the chronic stability of the device, enabling tracking of identical single‐neuron activity across the entire adult lifespan of the animal with high signal‐to‐noise ratios and minimal drift.

Encapsulation of neural probes in a soft matrix is another widely adopted strategy to alleviate mechanical stress caused by rigid implants. To address this, Park et al. developed a hybrid multifunctional probe encapsulated in a poly(acrylamide)‐alginate (PAAm‐Alg) hydrogel matrix, which closely matches brain tissue mechanics and reduces FBRs.^[^ ^39^ ^]^ Thermally drawn polymer‐based microfibers were integrated into the hydrogel matrix, forming a composite structure without compromising probe functionality (Figure 4b). The device incorporates a central polycarbonate (PC) optical waveguide clad with cyclic olefin copolymer (COC), flanked by three microelectrode arrays (80.0 ± 1.8 µm diameter, each with seven tin electrodes of 4.75 ± 2.22 µm) and three microfluidic channels (54.0 ± 2.1 µm inner / 115.4 ± 3.0 µm outer diameter), all aligned using a polymer guide fixture. Multiple hydrogels – PVA, alginate, PEGDA‐alginate, and PAAm‐Alg – were tested, but only PAAm‐Alg met the necessary criteria of high mechanical and chemical stability, softness, biocompatibility, and interfacial toughness (>150 J m^−2^ fracture toughness; >20 J m^−2^ interfacial toughness). Robust bonding between the hydrogel and functional fibers was achieved via surface amine functionalization, hydrogel dip coating, and UV crosslinking. A hydrogel thickness of 25 µm was selected to minimize device footprint while ensuring soft mechanical properties despite the underlying stiffer fibers. Finite element analysis showed that the fully swollen probe had a bending stiffness of 7 N m^−1^ – dramatically lower than that of stainless steel (8191 N m^−1^), silica (2150 N m^−1^), or PC (103 N m^−1^) – as the hydrogel effectively isolated mechanical stress across individual fibers. Furthermore, while soft probes typically require insertion aids, temporary dehydration of the hydrogel increased stiffness sufficiently for direct implantation, after which rehydration from surrounding tissue restored its original compliance. Chronic electrophysiological recordings in the ventral hippocampus were conducted for up to six months, capturing stable spontaneous neural activity and responses to optical stimulation. Initially, high noise levels and low SNRs were observed in the first two weeks post‐implantation, but both improved over time, indicating enhanced recording quality as tissue integration progressed.

Reducing the dimensions and mechanical rigidity of neural probes significantly enhances their biocompatibility. Luan et al. developed nanoelectronic thread (NET) probes using a minimally invasive implantation strategy for long‐term electrical recording in the brain.^[^ ^31^ ^]^ Two NET probe variants were fabricated: NET‐50, with a thickness of 1 µm and a width of 50 µm, containing eight electrodes arranged linearly; and NET‐10, with a thickness of 1.5 µm and a width of 10 µm, featuring four electrodes positioned on opposing surfaces. These ultrathin geometries substantially lowered bending stiffness to the 10^−15^ N·m^2^ range, comparable to cellular traction forces, enabling glial scar–free integration (Figure 4c). The probes were fabricated via photolithography using SU‐8 as the insulating layer and Pt or Au as the electrode material (100 nm thick). Due to their extreme flexibility, NET probes cannot penetrate brain tissue unaided. While conventional stiffening strategies or shuttle devices have typically exceeded 100 µm in size, the authors developed a novel delivery method employing carbon fiber and tungsten microwires with diameters as small as 7 µm, micromachined using a focused ion beam (Figure 4d). This allowed insertion with minimal acute damage (10 µm footprint). Chronic biocompatibility was assessed through in vivo two‐photon imaging via a cranial window. Although initial implantation caused transient blood‐brain barrier leakage, vascular and tissue integrity were fully restored within two months. Repeated recordings over four months in anesthetized mice showed high signal‐to‐noise ratios and stable action potential waveforms, confirming both functional and structural longevity of the probes.

Conventional intracranial implantation of neural probes typically requires craniotomy, which is highly invasive and can cause brain injury even before device placement. To address this, endovascular approaches have been explored for minimally invasive neural recording.^[^ ^130^, ^131^ ^]^ Wang et al. developed an ultraflexible, intravascular neural electrode (uFINE‐I) capable of recording electrophysiological brain signals via cerebral veins, thus avoiding direct brain penetration.^[^ ^125^ ^]^ The venous route was chosen due to its lower blood pressure relative to intracranial pressure, thereby reducing the risk of hemorrhage during vascular wall penetration. Sheep were used as animal models due to their intracranial vascular anatomy being comparable to that of humans. The uFINE‐I was specifically engineered for delivery through a minimally invasive system comprising a puncture microneedle, a microcatheter, and a guiding microwire (Figure 4e) A balloon catheter was also employed to facilitate positioning within the brain vasculature (Figure 4f). The device measured 50 mm in length, 5 µm in thickness, and 120 µm in width – dimensions optimized for the long navigation path from the jugular vein to the confluence of sinuses. The substrate was fabricated from polyimide for its mechanical robustness. The recording array consisted of 30 Ti/Au electrodes (5/100 nm thick), each 30 µm in diameter, spanning a linear region of ≈1.2 mm. Interconnects were composed of Ti/Au/Ti (5/100/5 nm thick), and electrode surfaces were coated with either iridium oxide or PEDOT:PSS to reduce impedance. Following implantation into the occipital lobe, the device successfully recorded both LFPs and single‐unit activity. Its functional performance was further validated by evoked responses to flickering visual stimuli, demonstrating the ability to capture both LFP and spike activity. Long‐term biocompatibility was assessed after 30 days of implantation through immunofluorescence and Hematoxylin and Eosin (H&E) staining, showing minimal glial encapsulation and evidence of vascular healing at the probe entry site.

The application of neural implants extends beyond the central nervous system to include peripheral targets. Carnicer‐Lombarte et al. developed an ultra‐conformable cuff electrode designed to enable sub‐nerve resolution for both recording and stimulation.^[^ ^126^ ^]^ This approach addresses two major limitations of peripheral nerve interfaces: the low selectivity of large area epineural electrodes and the high invasiveness of intraneural implants. The device was fabricated using 4 µm thick parylene‐C as the substrate, chosen for its exceptional flexibility and ability to conform tightly to peripheral nerves (Figure 4g). Standard photolithography was used to define Ti/Au interconnects (10/100 nm) and PEDOT:PSS microelectrodes (100 × 100 µm). Two additional larger electrodes were incorporated, one for whole‐nerve activity recording and the other serving as a ground. The system consisted of three cuffs, each specifically designed to match the dimensions of the median, ulnar, and radial nerves in the upper forelimb of rats (Figure 4h). To enhance mechanical stability and conformity, the cuffs featured gold‐reinforced anchoring tabs with locking holes. Electrophysiological performance was evaluated over a 21‐day period in awake, freely moving rats, during which stable action potential recordings with consistent spike amplitudes and signal‐to‐noise ratios were maintained. Principal component analysis (PCA) of the recorded spikes showed broad clustering, indicating activity from diverse axonal populations. Long‐term biocompatibility was assessed 28 days post‐implantation via immunohistochemical analysis of the radial nerve. For comparison, cuffs made of other biocompatible materials – including polydimethylsiloxane (PDMS) and polyethylene – were also tested. Immunostaining for α‐smooth muscle actin (αSMA) positive myofibroblasts revealed that the parylene‐C cuffs induced significantly less fibrotic encapsulation, confirming its superior performance as a substrate for chronic peripheral nerve interfacing.

#### 3.1.2 Material Innovations

The functional performance and in vivo stability of neural interfaces are critically influenced by the properties of the substrate materials.^[^ ^134^, ^135^, ^136^ ^]^ Serving as the structural basis for multi‐channel electrodes and integrated circuits, substrates must meet a complex set of requirements, including mechanical flexibility, chemical stability, electrical insulation, and biocompatibility. In long‐term implantation scenarios, substrates that exhibit mechanical properties similar to neural tissues (e.g., brain or peripheral nerves) are essential to minimize inflammatory responses and maintain a stable interface between electrodes and tissue. Furthermore, substrate transparency and permeability are increasingly important to enable integration with complementary modalities, such as optogenetics and drug delivery.^[^ ^137^, ^138^, ^139^ ^]^ Recently, beyond conventional planar polymer platforms, advanced substrate materials with enhanced structural conformity and multifunctional integration have emerged. In this section, representative materials, including fluorinated elastomers, hexagonal boron nitride (h‐BN), and elastic nanofiber‐based substrates, are introduced.

Achieving long‐term performance in neural interfaces requires balancing electrode density (i.e., spatial scalability) and tissue compatibility for long‐term implantation (i.e., temporal scalability), which has prompted the adoption of soft and biocompatible substrates. To address these challenges, Floch et al. proposed a multilayer high‐resolution neural probe using a photopatternable dielectric based on perfluoropolyether dimethacrylate (PFPE‐DMA), which supports the 3D stacking of nanoscale metal electrodes.^[^ ^38^ ^]^ PFPE‐DMA exhibits an exceptionally low Young's modulus (≈0.50 MPa), ≈10000 times lower than conventional dielectrics such as SU‐8 or PDMS, and maintains stable electrochemical impedance in phosphate‐buffered saline (PBS) for over one year. Notably, this material demonstrated significantly reduced astrocytic and microglial activation in both cortical and spinal cord implantation environments, surpassing polyimide and SU‐8‐based probes in minimizing immune responses, while preserving NeuN signal intensity. These findings indicate improved signal longevity and functional integration, particularly in mechanically constrained regions like the spinal cord. Leveraging its chemical orthogonality against organic and aqueous solvents, PFPE‐DMA is compatible with multilayer photolithographic processes. The material overcomes previous processing limitations of elastomers through nitrogen‐diffuser‐based photolithography and plasma surface modification. The fabricated structures exhibit exceptional conformability—capable of self‐wrapping around glass tubes—and maintain mechanical integrity under dynamic strain due to surface wrinkling behavior. The strong self‐adhesion of PFPE‐DMA prevents interlayer delamination and preserves structural integrity even after >20% compressive deformation. As shown in Figure 4i, a four‐layer high‐density electrode array was successfully implemented based on the low modulus and process compatibility of PFPE‐DMA. The resulting 64‐channel device stably recorded single‐unit activity for over ten weeks in both cortical and spinal regions, and behavioral assessments revealed no significant physiological perturbations compared to sham controls. These results underscore PFPE‐DMA's promise as a next‐generation substrate material for neural interfaces, enabling both material compatibility and functional scalability.

In high‐density multi‐channel neural interfaces, not only the mechanical compliance of the substrate but also its ability to insulate and shield against unwanted electrical signals (i.e., noise) becomes increasingly critical. Lee et al. introduced “NeuroWeb,” an ultrathin, flexible electrode array that integrates the resolution of implantable microelectrode arrays (iMEA) with the safety of surface electrode arrays (SEA).^[^ ^127^ ^]^ This platform features a ≈100 nm‐thick open‐lattice structure that fully conforms to the cortical surface and enables stable single‐neuron spike recording. A key innovation lies in the incorporation of h‐BN as the interlayer dielectric, which exhibits excellent insulation with no leakage current in PBS despite its nanometric thickness, while effectively shielding the electrodes from external electrical interference. The electrode configuration comprises Pt–Au–Pt multilayers and trilayer graphene interconnects, sandwiched by two h‐BN encapsulating layers. As shown in Figure 4j, this design enables electrical decoupling between closely packed electrodes, facilitating density scaling. Finite element modeling and adhesion testing confirmed that the thin h‐BN layer increases adhesion stress, allowing the structure to maintain intimate contact with complex surfaces without wrinkling. Furthermore, vertical stacking of the graphene/h‐BN configuration allowed for multilayer electrode arrays without increased leakage, supporting high‐fidelity signal transmission. Beyond electrical insulation, h‐BN provides mechanical compliance, adhesion, optical transparency, and vertical scalability, making it a strategic material for future ultrahigh‐density neural interface development.

As the intrinsic mechanical and electrical properties of materials increasingly dictate the performance of neural interfaces, fiber‐based substrates that offer stretchability, permeability, and biocompatibility have gained attention. Zhuang et al. demonstrated a liquid metal electrode array built on electrospun poly(styrene‐block‐butadiene‐block‐styrene) (SBS) nanofibers, offering mechanical softness, gas and moisture permeability, and chronic biocompatibility.^[^ ^128^ ^]^ The high porosity of SBS facilitates cell infiltration, promotes interfacial integration with surrounding tissue, and suppresses fibrotic encapsulation. As visualized in Figure 4k, silver patterns were first formed on wafer‐scale SBS nanofiber substrates, followed by the application of eutectic gallium–indium (EGaIn) to produce free‐standing micro‐liquid metal electrodes (µLMEs). A thin gold layer was deposited to enhance the spreading and pattern fidelity of the liquid metal. Importantly, the fabrication process was photolithography‐free, enabling scalability. The resulting substrate exhibited strain‐induced wrinkling behavior, conformal adhesion to complex surfaces like skin and brain, and sustained function under repeated bending, washing, and mechanical cycling. Immunohistochemical analysis revealed significant reductions in macrophage infiltration and fibrotic capsule thickness, confirming long‐term biocompatibility. This study highlights the potential of elastic nanofiber substrates to overcome the limitations of rigid or thick polymeric substrates, representing a promising strategy for next‐generation minimally invasive, high‐density neural interfaces.

Electrode materials play a pivotal role in neural interfaces by enabling both signal acquisition and electrical stimulation.^[^ ^140^, ^141^, ^142^, ^143^ ^]^ Beyond electrical conductivity, their mechanical compliance and biocompatibility must be precisely engineered, especially for high‐density, multi‐channel neural probes requiring electrode miniaturization, low impedance, and seamless integration with soft neural tissue. Conventional metal electrodes, despite their high conductivity, exhibit substantial mechanical mismatch with brain tissue, posing challenges for long‐term implantation. To address these limitations, recent advances have explored alternative materials such as conductive polymers, hydrogel‐based soft electrodes, and liquid‐metal systems, which maintain stable electrical performance while minimizing tissue damage.

Hydrogel‐based interfaces represent a promising class of multifunctional neural electrodes that integrate therapeutic capabilities. Qu et al. developed a monolithic microneedle array for hydrogel bioelectronics (hydroElex), composed of an interpenetrating network of PEDOT:PSS and N‐(3‐sulfopropyl)‐N‐methacroyloxyethyl‐N,N‐dimethylammonium betaine (DMAPS), which simultaneously enables neural signal recording and on‐demand drug release.^[^ ^129^ ^]^ The conductive hydrogel was fabricated via a single‐step UV polymerization, ensuring mechanical conformity with brain tissue. Depending on the DMAPS composition, the hydrogel exhibited conductivity ranging from 1.2 to 15.1 Sm^−1^ and a compressive modulus of 10 to 35 kPa, closely matching the mechanical properties of the brain. Internally, the hydrogel displayed a porous microstructure (Figure 4l), acting as a permeable scaffold that facilitates drug loading and metabolite diffusion. This architecture enabled selective drug release triggered by electrical stimulation while preventing passive leakage, thereby suppressing chronic immune responses. Even after 21 days post‐implantation, swelling ratio (124.3 ± 23.0%) and immunoreactivity remained comparable to sham controls. The microneedles were engineered with a height of ≈670 µm and a tip sharpness of 20.85°, allowing deep cortical insertion with minimal invasiveness. Electrochemical assessments showed stable charge storage capacity (35.1 mCcm^−^^2^) after over 100 CV cycles and maintained impedance in the 0.01–0.1 MΩ range at 1 kHz—suitable for LFP recordings. The scalable design was validated through multi‐channel array integration (Figure 4m), and drug release tests demonstrated an over 1000‐fold enhancement in release efficiency compared to passive diffusion, maintaining 78% cumulative release over 50 cycles. This monomaterial‐based closed‐loop interface highlights a promising platform for functionally integrated and biocompatible neural therapies.

In parallel, increasing demand for simultaneous electrophysiological recording and optical imaging has accelerated the development of transparent electrodes.^[^ ^144^ ^]^ Kim et al. introduced a fully polymer‐based PEDOT:PSS electrode array that satisfies both electrical and optical requirements without relying on metals.^[^ ^63^ ^]^ While metal electrodes offer excellent conductivity, their use in conjunction with two‐photon microscopy (2PM) can induce photothermal artifacts and fluorescence scattering, degrading signal fidelity. To overcome these issues, the authors employed a treatment protocol using formamide, phosphoric acid, and ethylene glycol to enhance the electrical performance of PEDOT:PSS, forming 20 × 20 µm^2^ electrodes and interconnects entirely from the polymer. Fabricated on a 25 µm PET substrate, the electrodes maintained flexibility and mechanical compatibility with the brain. Structural analysis (Figure 4n) confirmed conformal contact between the patterned PEDOT:PSS film and cortical tissue. Electrochemical characterization showed an impedance of 45.8 kΩ at 1 kHz and SNR on par with Pt‐black electrodes. Optically, the FPE‐PEDOT film—treated with formamide, phosphoric acid, and ethylene glycol—achieved 73% average transmittance in the visible range, outperforming traditional ITO, tri‐layer graphene, and Pt films. In vivo experiments demonstrated simultaneous 2PM calcium imaging and electrophysiology in the mouse V1 cortex with negligible optical noise, establishing its potential as a core platform for multimodal neural research involving fMRI, optogenetics, and high‐resolution imaging.

More recently, liquid metal‐based stretchable electrodes have gained attention as a highly promising material platform that combines extreme flexibility with high electrical conductivity.^[^ ^145^, ^146^, ^147^, ^148^, ^149^, ^150^, ^151^ ^]^ Kwon et al. developed a fully integrated wireless neural recording system by directly printing the electrodes, interconnects, and power supply onto the curved surface of a mouse skull.^[^ ^49^ ^]^ EGaIn (75.5% Ga, 24.5% In) was employed as the electrode material, precisely printed onto a 1 µm parylene‐C substrate at 5 µm resolution, followed by electrochemical deposition of Pt‐black to enhance electrical properties. The resulting PtB/EGaIn electrodes achieved an impedance of 387.5 kΩ at 1 kHz—≈3× lower than uncoated EGaIn—and an area‐specific impedance of 15.2 Ω·mm^2^, one‐third that of platinum. Mechanically, the PtB/EGaIn electrodes exhibited an ultra‐low Young's modulus of 211 kPa, representing a 10⁶‐fold reduction compared to platinum and a 10⁴‐fold reduction compared to PEDOT:PSS, thereby offering high compliance with brain tissue. Notably, the intrinsic self‐healing property of the liquid metal allowed the electrodes to recover their conductivity within 5 s after temporary disconnection under pressure, with electrical stability maintained over 200 cycles in PBS. The EGaIn interconnects were also directly printed to form 16‐channel connections with a minimum linewidth of 5 µm, integrating seamlessly with cortical electrodes, a Wi‐Fi module, and a battery into a single conformal platform. The system successfully recorded single‐neuron spikes and LFPs wirelessly from both the motor cortex and hippocampus, with PCA‐based clustering confirming high‐resolution signal acquisition. Figure 4o illustrates a fully conformal, high‐density neural interface directly printed onto the skull, highlighting the practical feasibility of soft, fully implantable platforms for chronic neural monitoring and closed‐loop neuromodulation in freely behaving animal models.

Despite the mechanical advantages and high conductivity of EGaIn, its long‐term chronic application in neural interfaces requires careful attention to its electrochemical stability. Upon exposure to air or biological fluids, EGaIn rapidly forms a native gallium oxide (Ga_2_O_3_) layer, which provides mechanical stability. However, when applied in neural interfacing devices, EGaIn electrodes in aqueous environments face major bottlenecks in terms of electrochemical stability and electrical conductivity. Specifically, EGaIn electrodes can exhibit broad and pronounced redox current responses that obscure the electrochemical signals of target neurons and alter their profiles due to modulation of the liquid metal's surface tension. In addition, the formation of a native Ga_2_O_3_ layer with insulating properties can hinder electron transfer of target biomolecules at the electrode–electrolyte interface, increasing the impedance and can degrade over time under physiological stress. Chronic in vitro studies have revealed progressive thickening of this oxide layer, leading to increased interfacial resistance and potential ion leaching, particularly of Ga^3^⁺ ions, which may induce cytotoxic effects if unmitigated. Therefore, creating electrochemically stable liquid metal electrodes for long‐term neural interfacing can be achieved by employing multiple material‐level strategies.

First, encapsulation of EGaIn within biocompatible polymers such as PDMS or Parylene‐C physically isolates the liquid metal from surrounding tissues, preventing bulk leakage or electrochemical degradation, while preserving mechanical flexibility and stability. In neural interfaces, this has enabled the formation of high‐density EGaIn microelectrode arrays with stable performance under dynamic strain. Kim et al. demonstrated a high‐resolution multielectrode array that can precisely record electromyography (EMG) signals by penetrating through the epidermis to target the muscle. Passivation of the sidewalls of the liquid metal pillars enabled the electrical insulation, providing accurate and reliable EMG signal acquisition with minimal signal distortion for over 25 days.^[^ ^119^ ^]^

Second, integrating conductive materials such as metal nanoparticles and carbon nanomaterials onto the EGaIn surface has been shown to improve its electrochemical stability under aqueous conditions. Chung et al. developed an artificial retina with 3D liquid‐metal microelectrodes using EGaIn as the pillar material. In this design, Pt nanoclusters were coated on the tips of the 3D microelectrodes, the stimulation sites interfacing with neural tissues. This strategy demonstrated good biocompatibility, as evidenced by a 5‐week implantation study showing no significant deformation or cytotoxic effects in the retinal tissues.^[^ ^120^ ^]^ Chemical passivation with conductive polymers at the electrode‐tissue interface has also proven essential for electrochemical stability.^[^ ^152^ ^]^ For example, ultra‐thin polymer coatings such as poly(3,4‐ethylenedioxythiophene) (PEDOT) can suppress oxidation while enhancing charge injection capacity. Lim et al. demonstrated that PEDOT‐coated EGaIn electrodes exhibited a ≈99% reduction in gallium ion release and sustained a >1000‐fold increase in charge storage capacity compared to uncoated controls, while enabling stable in vivo neural recordings over several weeks.^[^ ^153^ ^]^

Nanocomposite approaches, such as wrapping EGaIn particles with reduced graphene oxide, have also improved oxidation resistance.^[^ ^154^ ^]^ Lee et al. developed functionalized EGaIn electrodes in which reduced graphene oxide is self‐assembled around EGaIn particles to form core–shell structures. These reduced‐graphene‐oxide assembled EGaIn (REG) electrodes exhibited robust interfacial adhesion with the underlying current collector, preventing delamination under repeated stretching, and demonstrated excellent electrochemical stability. Moreover, by decorating the REG shells with noble metal nanoparticles such as Pt or Au, the electrodes achieved enhanced electrocatalytic activity and enabled simultaneous detection of multiple biomolecules (ascorbic acid, dopamine, and uric acid) as well as enzymatic glucose sensing, all while maintaining stable performance under mechanical deformation.

While structural and material innovations in devices have significantly improved biocompatibility and reduced tissue damage, the implantation process itself still poses considerable challenges that can compromise device performance. In particular, the compliant nature of ultrasoft probes often prevents them from penetrating neural tissue unaided, leading to the development of various insertion strategies using temporary stiffening aids such as microneedle shuttles, capillary‐bound microwires, or bioresorbable polymer coatings.^[^ ^155^, ^156^ ^]^ However, the insertion tools—often larger and stiffer than the probes themselves—can induce acute tissue damage and inflammation, potentially leading to early signal degradation. Furthermore, during and after implantation, mechanical mismatch, micromotion, and chronic immune responses can result in electrode encapsulation by glial cells, delamination, material corrosion, or increased impedance, all of which contribute to signal attenuation or complete recording failure over time. To mitigate these issues, recent approaches have explored not only softer, tissue‐like geometries that promote seamless integration and reduce gliosis, but also biologically inspired strategies that facilitate tissue ingrowth into the device.

For example, NeuroRoots, a bio‐inspired neural interface, emulates the axonal bundle architecture of the brain by distributing multiple ultrathin electrode filaments that mimic natural axon fascicles.^[^ ^218^ ^]^ This design allows the electrodes to move flexibly with the surrounding tissue, thereby reducing acute insertion damage and minimizing chronic tissue stress. In freely moving rats, 32 densely packed channels were stably implanted, demonstrating minimal gliosis and long‐term recording stability. The combination of mechanical compliance, bio‐mimetic geometry, and high‐density channel distribution enables NeuroRoots to maintain high‐fidelity neural signals while significantly reducing tissue disruption. These techniques aim to stabilize the neuron‐electrode interface and maintain recording fidelity over extended periods. A more systematic understanding of electrode failure mechanisms, including mechanical, chemical, and biological factors, is essential to guide the next generation of durable, chronic neural interfaces (**Table** **3**).

**Table 3:** Mechanical, optical, electrical, and ionic properties of representative materials. Ionic conductivity values were measured using electrochemical impedance spectroscopy (EIS).

	Material	Elastic Modulus	Transmittance	Impedance (at 1 kHz)	Ionic Conductivity (EIS)	Reference
Substrate/Packaging Materials	Parylene C	2.46 GPa	87%	Non‐conductive	N/A	[157, 158]
	Polyimide	2.5 GPa	71%			[159]
	h‐BN	865 Gpa	96%			[127]
	SU‐8	2.0 GPa	97%		≈1 × 10^−9^ Sm^−1^ ^a)^	[38]
	SEBS	2.83 MPa	90%		9.44 × 10^−9^ Sm^−1^ ^b)^	[160, 161, 162]
	PDMS	100 kPa to 3 Mpa	90%		4.45 × 10^−8^ Sm^−1^ ^b)^	[163, 164]
	Fluorinated Elastomers	0.50 Mpa	N/A		≈3 × 10^−9^ Sm^−1^ ^a)^	[38]
Electrode Materials	Au	78 GPa	Opaque	5.4 Ω⋅cm^2^	N/A	[31, 159]
	Pt	170 GPa		1.77 Ω⋅cm^2^		[124, 165]
	Liquid Metals	211 kPa		0.152 Ω·cm^2^		[49]
	PEDOT:PSS(Clevios PH‐1000)	≈1 MPa	73%	11.45 Ω·cm^2^		[63, 129]
	Conductive hydrogels ^c^	10‐35 kPa	N/A	0.22 Ω·cm^2^	1.2 –15.14 Sm^−1^	[129]

^a)^ At 37 °C after aging in 1× PBS;

^b)^ At 23 °C after aging in 1× PBS;

^c)^ Hydrogel composition: PEDOT:PSS 0.7% (w/v) + DMAPS 20% (w/v)).

### 3.2 Data Analysis and Interpretation

Multi‐channel neural interfaces offer a distinct advantage over single‐channel systems by enabling the acquisition of high‐dimensional neural signals across multiple spatial regions. The multiplicity in both size and dimension unlocks a wide range of analytical techniques. In particular, it allows inter‐channel comparative analyses, which can reveal functional interactions among different brain regions. Such capabilities enable various novel experiments, including single‐neuron tracking and spatiotemporal correlation analyses. Furthermore, machine learning techniques can be employed to uncover complex and nonlinear relationships that lie between the data. This section reviews representative analytical techniques used to analyze and interpret the signals acquired from multi‐channel neural interfaces.

#### 3.2.1 Functional Connectivity

**Figure 5:**
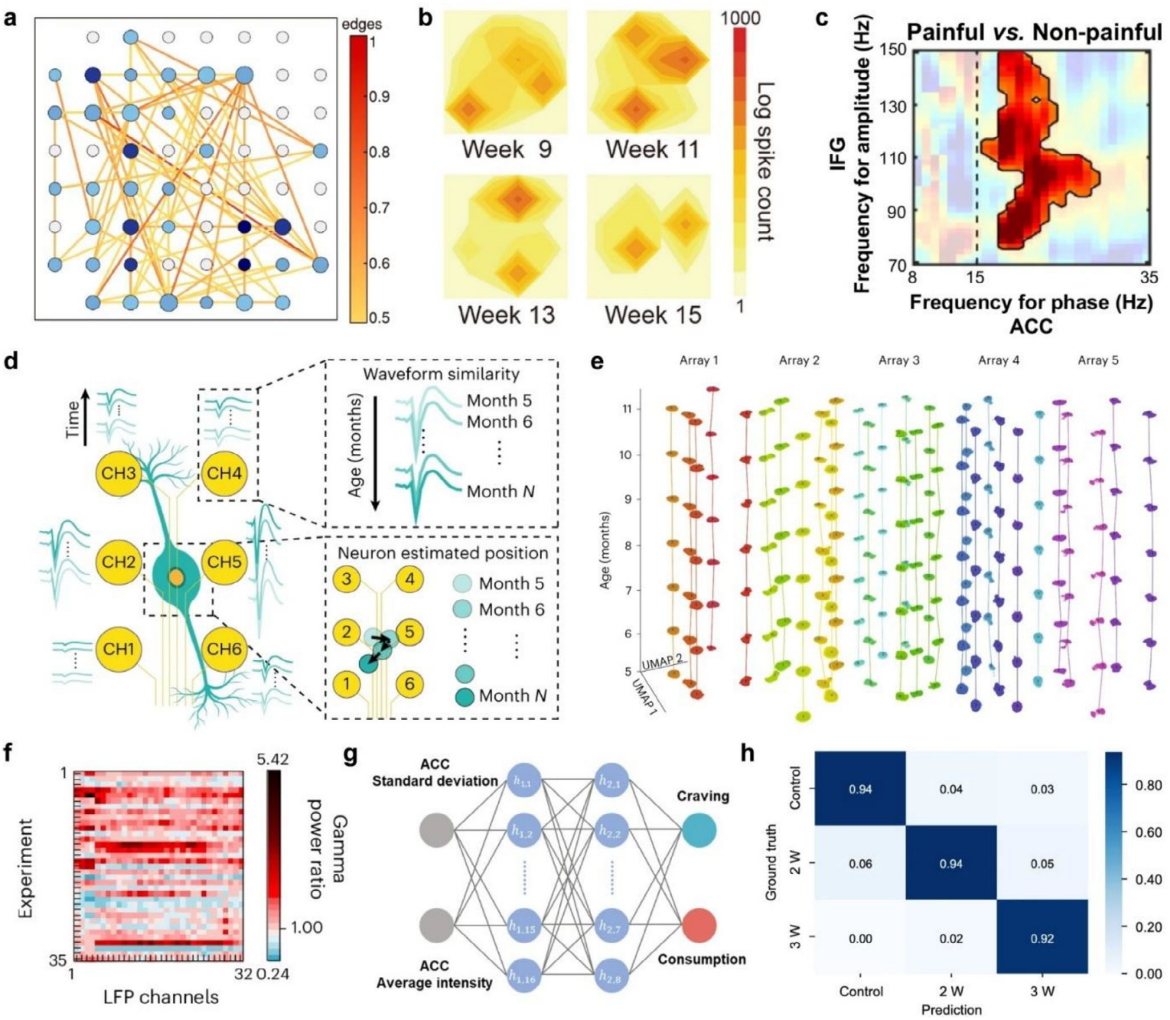
Methods of data analysis and interpretation acquired from multi‐channel neural interfaces. a,b,c) neural connectivity analysis: a) Neural network map based on synchronization of the neurons. Each node represents an electrode of the 3D MEA, while the lines connecting the nodes indicate occasions where the synchronization score exceeds 0.5, and the color of the lines represents the magnitude of neural connectivity. Reproduced with permission.^[^ ^14^ ^]^ Copyright 2025, Springer Nature. b) Contour plot showing the regional firing rates of RGCs across the retinal surface. Reproduced with permission.^[^ ^173^ ^]^ Copyright 2024, John Wiley and Sons. c) PAC comodulogram visualizing the difference between PAC values of IFG and ACC regions in an individual subject to a painful stimulus, and the same individual subject to a non‐painful stimulus. Reproduced with permission.^[^ ^174^ ^]^ Copyright 2024, Springer Nature. d,e) long‐term tracking: d) Schematics showing the mechanism of long‐term single‐neuron tracking. e) Time evolution of the single‐unit spikes characterized by the UMAP algorithm. Reproduced with permission.^[^ ^124^ ^]^ Copyright 2023, Springer Nature. f,g,h) Machine learning: f) Scalogram showing the gamma power ratio acquired from all experiments and channels. Reproduced with permission.^[^ ^59^ ^]^ Copyright 2024, Springer Nature. g) Schematics of the DNN model architecture for the classification of craving and consumption behaviors of monkeys. Reproduced with permission.^[^ ^59^ ^]^ Copyright 2024, Springer Nature. h) Confusion matrix of the prediction results and the ground truth between the control, 2‐week neuroma group, 3‐week neuroma group. Reproduced with permission.^[^ ^178^ ^]^ Copyright 2025, Springer Nature.

Since the multi‐channel neural interfaces enable simultaneous recordings from spatially distributed spots within the neural tissues, they are particularly beneficial for investigating neural function that operated under neural circuit level activations. This is essential for studying neural connectivity and for elucidating the underlying mechanisms of neurophysiological development and neurological disorders.^[^ ^166^, ^167^, ^168^, ^169^ ^]^ In such investigations, various mathematical methodologies are employed to characterize neural connectivity. Representative approaches include phase synchronization, phase locking, and phase‐amplitude coupling (PAC), each of which captures distinct aspects of the temporal and spectral relationships between neural signals. The derived connectivity patterns can subsequently be visualized using multiple modalities, including network maps, rose plots, and comodulograms, thereby facilitating intuitive interpretation of complex dynamic interactions.^[^ ^170^, ^171^, ^172^ ^]^ For example, Kim et al. developed a 3D multielectrode array to record from multiple spatial spots within brain organoids. Two primary analyses were employed on the neural signals acquired using the device.^[^ ^14^ ^]^ One analysis is the synchronization score, which quantifies the functional connectivity developed within brain organoids. For calculation, the timepoints of spikes from 2 individual electrodes were compared, which yielded the temporal difference between the spikes. Then, the degree of proximity between the spikes was computed and shown as the synchronization score, ranging from 0 to 1, with higher values indicating stronger synchrony. Based on synchronization score, another analysis used in this study was identifying the neural community. To compute the neural community, each electrode in the array was first defined as a node, and a line was created if the synchronization score between two nodes exceeded 0.5. Modularity was used as a parameter to designate the degree of complexity of the line between the nodes, and Louvain algorithm was applied to iteratively group nodes to maximize modularity. The resulting group of nodes that showed the highest modularity, which indicates the most complex circuitry, was designated as a neural community. These results were visualized as a neural network map (**Figure** **5**a), providing insight into the functional structure of the neural network. Furthermore, spatially outspread multi‐channel neural interfaces facilitate investigating neural activities across different cell layers.^[^ ^173^ ^]^ Lee et al. observed functional properties and connectivity of retinal ganglion cells (RGCs) of retinal organoids over time using multi‐electrode arrays. Figure 5b visualizes regional firing rates of RGCs across the retinal surface, which offers chronological information on the growth of RGCs. Additionally, the authors investigated the existence of neural connectivity between cell layers developed within retinal organoids using spatially distributed electrodes contacted with two different cell types. They conducted phase locking analysis, which compares two signals from different regions by assessing whether spike timings in one signal were aligned with specific phases of another signal. The existence of phase locking between signals of different spots indicates robust functional connections. When the group examined phase locking between RGCs and retinal progenitor cells (RPCs) in the gamma oscillations (28–100 Hz), no significant synaptic connectivity was observed, indicating incomplete resemblance between retinal organoids and retina regarding functionalities.

Another important technique that is used to evaluate the functional connectivity of neurons is the PAC. Fundamentally, PAC is a phenomenon that occurs between two waves with different frequencies, where the amplitude of the higher‐frequency signal is affected by the lower‐frequency signal. As a means of analysis, PAC finds the degree of coupling between two signals. For example, Tan et al. conducted PAC to investigate pain‐related brain dynamics using neural signals from the anterior insula, anterior cingulate, amygdala, and inferior frontal gyrus recorded by multi‐electrode interfaces.^[^ ^174^ ^]^ A representative example of a comodulogram, which examined the PAC between the anterior cingulate and inferior frontal gyrus, is shown in Figure 5c. PAC results indicated that high‐gamma amplitude of the inferior frontal gyrus was locked to beta phase in the anterior cingulate at the painful condition, providing insight into neural dynamics regarding pain. Furthermore, several papers conducted PAC analyses to examine neural responses to external stimuli at the brain circuit level, showing the modulation of coupling strength.^[^ ^175^, ^176^, ^177^ ^]^ These observations offer valuable insights into how neural dynamics are altered by diseases or therapeutic interventions, which contribute to a deeper understanding of neural networking mechanisms and support the development of effective neurotherapies.

#### 3.2.2 Single‐Neuron Tracking

Beyond functional connectivity analysis, multi‐channel interfaces also support longitudinal single‐neuron tracking, which is possible due to the ability of multi‐channel devices to record spikes of a single neuron from multiple electrodes. For single‐neuron tracking, a fundamental analytical technique is spike sorting, which is commonly implemented using clustering algorithms based on principal component analysis (PCA).^[^ ^178^ ^]^ In this approach, PCA is applied to extract orthogonal axes, which are referred to as “principal components”, that capture the maximal variance in the recorded spike waveforms. The original data are then projected onto these principal components to facilitate dimensionality reduction and enhance separability among spike shapes. This transformation enables more effective clustering of spikes originating from distinct neurons, thereby allowing for the identification and classification of individual neuronal activity. For example, Zhao et al. fabricated 16‐channel and 32‐channel mesh electrodes to track individual neurons throughout the adult lifespan of a mouse.^[^ ^124^ ^]^ Figure 5d describes how spikes from a single neuron can be recorded by multiple electrodes, which enables the authors to estimate the position of the neuron. Given the average neuron displacement of 2.61 µm over the 7‐month recording, the 15 µm‐spaced electrodes could reliably track single units without any significant drift of electrodes. Additionally, the waveforms detected by electrodes were analyzed by investigating various wave features such as phase and amplitude; therefore, the authors could determine that recorded signals from an electrode are from an identifiable neuron. The uniform manifold approximation and projection (UMAP) sorting showed nearly constant positions and good separation of spikes throughout the recording, as shown in Figure 5e. Similarly, Le Floch et al. applied similar techniques using novel 3D neural probes to track single neurons for up to 10 weeks.^[^ ^38^ ^]^ Also, Steinmetz et al. deployed 10240 probes into a mouse, and incorporated special algorithms to account for the motion of the probes after insertion, such as the drift.^[^ ^179^ ^]^ To determine whether the firings were from a single neuron, the mice were presented with 112 natural images to induce the firing of neurons. A five‐time repetition of the experiment allowed comparison of the firings of the neurons, which in turn allowed the group to determine whether the firings were from the same neurons by investigating the signals from different channels and those from different repetitions, thereby fingerprinting the neurons. This was repeated for 15 days, after which the fingerprints from neighboring days were compared to investigate whether they had closer coefficients of correlation than the fingerprints from the neighboring probes. If the neighboring days exhibited a higher correlation, the firings were considered to be from a single neuron. The idea of clustering the spikes to determine a single neuron is also shown in other studies. Park et al. conducted multiple studies that involved single‐neuron tracking using multi‐channel neural interfaces.^[^ ^39^, ^180^ ^]^ The detected spikes were sorted based on various aspects of the waveforms, including the amplitude, shape, and PCA clustering, which were tracked for 6 months. The clusters acquired at different timepoints were found to be largely overlapping, which suggested that the spikes in each cluster were from the same neuron.

#### 3.2.3 Machine Learning

Machine learning is also extensively used to investigate the signals recorded from multi‐channel neural interfaces. Given that neural activity contributes to a wide range of physiological and behavioral processes, including behavior, emotion, pathological states, and even tissue generation, decoding these signals holds immense potential not only for advancing our understanding of such phenomena, but also for enabling targeted modulation, particularly in the context of disease treatment.^[^ ^181^, ^182^, ^183^ ^]^ However, due to the intrinsic complexity and high dimensionality of neural signals, conventional signal processing techniques are often insufficient for capturing the underlying patterns. This necessitates the application of computational models, such as machine learning algorithms, that can be trained to identify and interpret latent relationships embedded within neural activity. The usage of machine learning can vary from simple classification to prediction.^[^ ^184^, ^185^, ^186^, ^187^, ^188^ ^]^ Oh et al. employed a convolutional neural network (CNN) and a deep neural network (DNN) to classify the eating behaviors of monkeys.^[^ ^59^ ^]^ With a 32‐channel neural probe, LFP and acceleration signals (ACC) were recorded from a monkey for over 1 month. From the LFP, the gamma band (35–50 Hz) was extracted and processed into scalograms and band power (Figure 5f), and these features were used as inputs for the CNN. From the ACC, average intensity and standard deviation were extracted and used as input into the DNN, as shown in Figure 5g. The two networks were concatenated to form a complex neural network. This model classified the eating behavior of a monkey into craving, seeking, and consumption with an accuracy of 86.33%. In another example, Ramezani et al. used multi‐channel graphene electrodes to record neural signals as well as calcium signals in layer 2/3 of the visual cortex of a mouse simultaneously.^[^ ^102^ ^]^ Simultaneously detected two types of signals enable to predict calcium signals based on neural signals. The neural signals were first filtered into various frequency ranges, from delta to multi‐unit activity (MUA). Those signals were then used as inputs to the neural network model, customized to include a linear hidden layer, a bidirectional long short‐term memory (BiLSTM) layer, and a linear readout layer. To predict the single‐cell calcium signal, Gaussian process factor analysis (GPFA) was used to reduce the dimensions of the features, which were fed into the three‐layer neural network model to predict 8‐dimensional time‐series latent variables. These predicted variables were converted back to represent calcium signals. Aside from neurological research, machine learning techniques can also be employed for disease monitoring. In a study conducted by Wang et al., a multi‐channel neural interface was fabricated for early diagnosis and management of neuroma by delivering electrical stimulation.^[^ ^189^ ^]^ Neural signals during a treadmill exercise were recorded from a rat and used as inputs for separate uses of 1‐dimensional convolutional neural network (1D‐CNN). From the evoked neural signals, the progression of neuroma was classified into control, 2 weeks into neuroma, and 3 weeks into neuroma with a maximum of 94% accuracy, as shown in Figure 5h.

Likewise, multi‐channel neural interfaces provide powerful opportunities to explore a wide range of neural phenomena, from functional connectivity and single‐neuron tracking to disease monitoring. With the advancement of measuring devices and analytical methodologies, we can expect the discovery of complexities of brain function that can aid in the development of neuromodulation strategies for clinical applications. While the analytical techniques described above, such as machine learning–based decoding and multivariate signal analysis, are effective under the assumption of reliably recorded multi‐channel signals, it is important to recognize that as channel count and interconnect density increase, the potential for electrical crosstalk via capacitive coupling also rises.^[^ ^190^ ^]^ In high‐density neural arrays, the reduced spacing between interconnects intensifies capacitive coupling, leading to signal attenuation, degraded signal‐to‐noise ratio (SNR), and potential misclassification during spike sorting or neural decoding. To address this bottleneck, two major solution strategies have been explored.

First, hardware and design‐based approaches have been proposed to mitigate electrical crosstalk, including strategies such as optimizing electrode spacing, integrating shielding layers, and improving interconnect layouts. Among these, a representative solution is the development of locally shielded electrode architectures in a coaxial geometry.^[^ ^219^ ^]^ In this design, each recording site is surrounded by a conductive shielding layer fabricated through micro‐ and nanofabrication processes, analogous to the structure of a coaxial cable. This architecture significantly suppresses capacitive coupling between adjacent interconnects, which is a primary source of crosstalk in high‐density arrays. As a result, the shielded arrays demonstrated at least a 400‐fold improvement in effective spatial density compared to unshielded electrodes, enabling reliable multi‐site recording without the need for post‐hoc spike sorting. This work highlights how hardware‐level innovations can fundamentally enhance signal fidelity and scalability in high‐density neural recording systems.

Second, computational and signal‐processing approaches aim to separate mixed signals during downstream analysis. Common strategies involve independent component analysis (ICA), blind source separation, and machine learning–based spike deconvolution. As a representative case, Leibig et al. applied convolutive ICA (cICA) to high‐density microelectrode array recordings and showed that this algorithm could reliably disentangle overlapping spikes, achieving high recovery rates and low error levels even under significant cross‐talk conditions.^[^ ^220^ ^]^

Together, these two strategies—hardware‐level suppression and computational disentanglement—highlight complementary pathways for overcoming the limitations imposed by high‐density integration in multi‐channel neural interfaces. A more thorough understanding and systematic modeling of crosstalk is essential for maintaining fidelity in advanced neural decoding pipelines.

### 3.3 Applications in Brain‐Machine Interface

Recent advances in structural and material engineering have enabled multi‐channel neural interfaces to record high‐fidelity neural signals with unprecedented spatial and temporal resolution. These high‐quality signals, captured across multiple channels, have facilitated the development of sophisticated analytical approaches for interpreting neural data.^[^ ^191^, ^192^, ^193^, ^194^ ^]^ In particular, the integration of machine learning techniques has significantly enhanced the efficiency and accuracy of neural signal decoding. As a result, researchers can now gain deeper insights into the functional roles and connectivity of neural circuits. Although many BMI applications have been successfully demonstrated using conventional interface designs, the integration of structural and material innovations significantly enhances signal fidelity, stability, and resolution—key factors in improving decoding precision and enabling more robust, closed‐loop systems. This progress has opened up a broad range of brain‐machine interface applications, from restoring impaired brain functions through targeted stimulation to developing closed‐loop BMI systems for real‐time disease monitoring and intervention. Moreover, these technologies are being explored for use in immersive environments such as virtual and augmented reality, further expanding the potential of BMIs beyond clinical contexts.^[^ ^195^, ^196^, ^197^, ^198^, ^199^, ^200^, ^201^, ^202^, ^203^ ^]^

In this section, we discuss the applications of BMIs across the translational spectrum, from cutting‐edge invasive multi‐channel interfaces to non‐invasive and commercially available devices. By framing BMI applications as a continuum from experimental technologies to clinically deployable systems, we highlight how high‐resolution signal acquisition, machine learning‐based analysis, and device stability converge to enable restorative, therapeutic, and augmented technologies.

#### 3.3.1 Restorative and Assistive BMI

**Figure 6:**
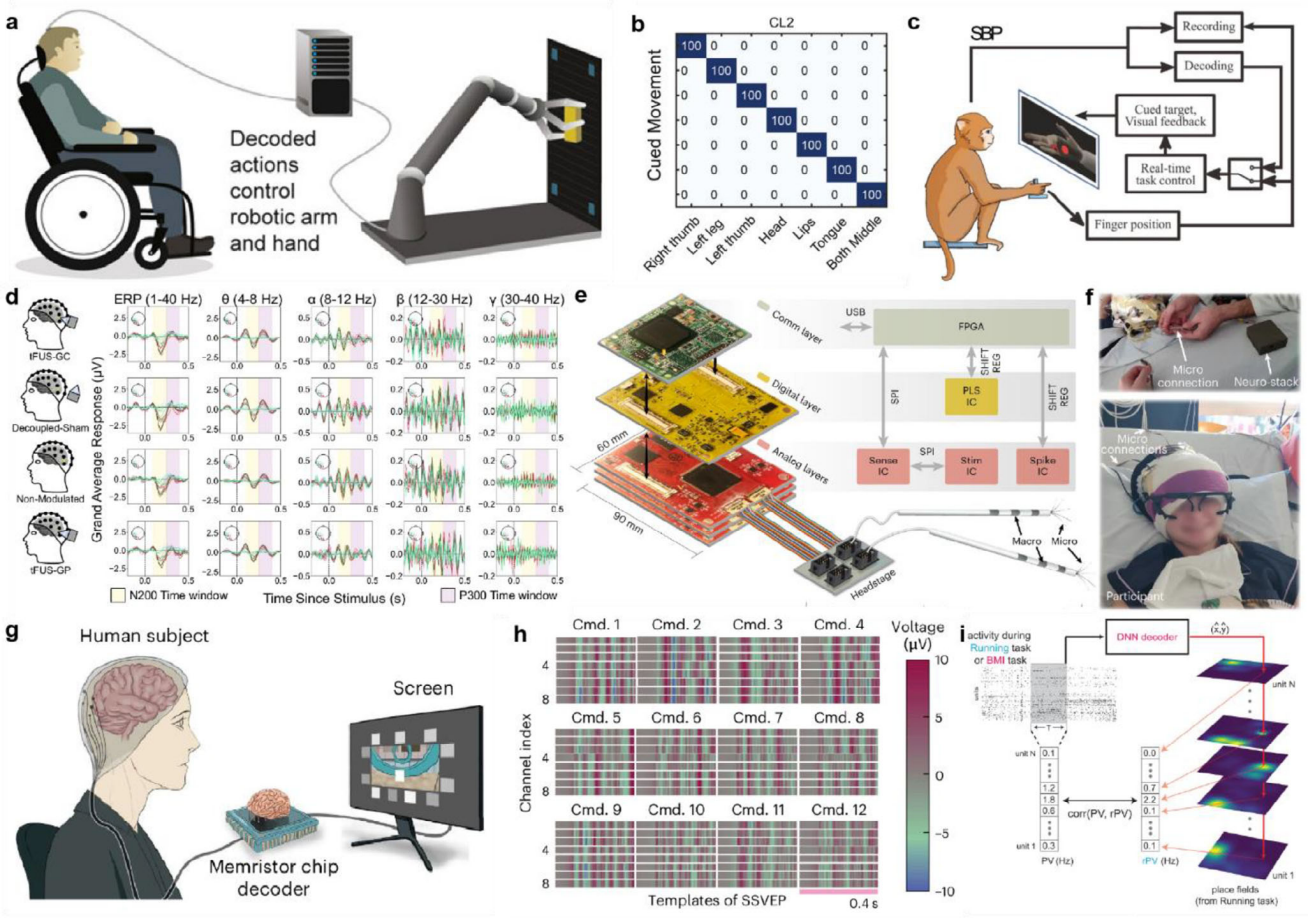
Representative approaches and applications in brain‐machine interfaces. a) A schematic of the BMI system for controlling the robotic arm and hand. b) Single‐session trial‐level confusion matrix of imagined actions. Reproduced with permission.^[^ ^208^ ^]^ Copyright 2025, Elsevier. c) Experimental setup of controlling the virtual finger. Reproduced with permission.^[^ ^209^ ^]^ Copyright 2022, Springer Nature. d) Electrode responses for theta, alpha, beta, and gamma frequencies. Reproduced with permission.^[^ ^212^ ^]^ Copyright 2024, Springer Nature e) Neuro‐stack device image. f) The Neuro‐stack is connected to micro‐electrodes in the participant. Reproduced with permission.^[^ ^213^ ^]^ Copyright 2023, Springer Nature. g) Schematic of the experimental setup of BMI for controlling drone. h) Templates of SSVEP from 12 drone commands. Reproduced with permission.^[^ ^214^ ^]^ Copyright 2025, Springer Nature. i) Comparison of the spiking activity at a given location. Reproduced with permission.^[^ ^215^ ^]^ Copyright 2023, American Association for the Advancement of Science.

Restorative and assistive BMIs function as auxiliary systems that supplement or replace certain body functions, while also offering the potential to induce recovery of impaired brain circuits through feedback stimulation during long‐term repetitive training.^[^ ^204^, ^205^ ^]^ O'Doherty et al. developed a closed‐loop BMI in which neural signals recorded from the motor cortex are decoded to control a virtual arm, and sensory feedback from the virtual arm is encoded into the brain.^[^ ^206^ ^]^ A multielectrode array consisting of 16 nanowires is implanted into the primary motor cortex (M1) and primary sensorimotor cortex (S1). The virtual‐reality arm or a computer cursor was controlled by motor commands decoded from ensemble neural activity in M1, measured at the moment the monkey moved a joystick. Movement intentions are decoded using an unscented Kalman filter, based on firing rates computed over 50 ms windows. Sensory information is delivered via intracortical microstimulation (ICMS) through nanowires implanted in S1. The experimental conditions included three types of artificial textures delivered via ICMS: a Rewarded Artificial Texture (RAT), which consisted of 400 Hz pulse trains presented in 5 Hz packets; an Unrewarded Artificial Texture (UAT), with 200 Hz pulse trains in 10 Hz packets; and a Null Artificial Texture (NAT), in which no stimulation was applied. With repeated trials, the monkey spent more time interacting with the RAT, confirming that sensory feedback delivered to S1 influenced motor activity in M1. In a follow‐up study by the same group, a bimanual arm movement BMI was developed using a 768‐channel microwire array.^[^ ^207^ ^]^ Furthermore, Natraj et al. demonstrated long‐term stable control of a robotic arm in a tetraplegic participant, using imagined movement signals recorded from the sensorimotor cortex with a 128‐channel electrocorticography (ECoG) grid (**Figure** **6**a).^[^ ^208^ ^]^ During trials involving 30 imagined actions, signals were filtered into the delta (−0.5–4 Hz), beta (12–30 Hz), and high gamma (70–150 Hz) bands. Power features from each band were extracted and decoded using the Refit‐Kalman Filter, which was made available as open‐source software. By sampling representational plasticity and drift across days, high‐degree‐of‐freedom robotic arm control was achieved without updating the decoder (Figure 6b). Willsey et al. further succeeded in decoding rapid and realistic finger movements by using spike‐band power features from M1, recorded through a 96‐channel Utah array (50–64 channels used), and decoded via the Refit‐Kalman Filter (Figure 6c).^[^ ^209^ ^]^

Beyond replacing motor functions, BMI research has also focused extensively on assisting a range of interaction modalities, including electric wheelchair navigation, cursor control, and speller input for communication.^[^ ^210^, ^211^ ^]^ Kosnoff et al. investigated event‐related potentials (ERPs) elicited by visual stimuli using a 64‐channel EEG cap equipped with 62 active electrodes (Figure 6d).^[^ ^212^ ^]^ Specifically, they analyzed changes in N200 power—defined as the average power within the 100 to 250 ms post‐stimulus time window—when a flashing line passed over the key that the participant was staring at with the intent to select. In particular, when transcranial‐focused ultrasound stimulation was applied to the V5 region of the visual cortex for 1 s per trial, a significant amplification in N200 power was observed, which led to a decrease in errors in performing the BMI task. In addition, target classification was performed using the N200 component induced by visual stimulation, and a custom Bayesian decoding algorithm was designed to support this purpose with improved reliability and specificity.

#### 3.3.2 Therapeutic BMI

Closed‐loop BMIs represent a promising platform for disorders requiring real‐time diagnosis and intervention, such as epilepsy and chronic pain. Sun et al. developed a closed‐loop therapeutic BMI that delivers either optogenetic stimulation or deep brain stimulation (DBS) to the prelimbic prefrontal cortex (PL‐PFC) when the LFP power recorded from the primary somatosensory cortex (S1) and the anterior cingulate cortex (ACC) of a chronic pain rat model exceeds a predefined threshold.^[^ ^216^ ^]^ LFP signals are recorded using two 3‐channel silicon probes inserted into the S1 and ACC, respectively. The power of the LFPs, calculated across low gamma, high gamma, and ultrahigh frequency bands, was fed into a state‐space model. When the z‐score of the extracted signal, relative to the baseline activity, exceeded a threshold of 3.38, the event was classified as a candidate pain signal. At this stage, a decoder based on the cross‐correlation function was implemented to ensure that stimulation was only triggered when similar dynamic changes in neural activity occurred simultaneously in both the S1 and ACC regions. The same research group subsequently developed a closed‐loop therapeutic BMI system for chronic pain treatment and constructed a functional prototype with potential for clinical translation and commercialization.^[^ ^217^ ^]^ The number of channels was expanded from the original 6 to 32, and the z‐score was computed based on ensemble spike counts derived from neural recordings, rather than from LFP power. Furthermore, Topalovic et al. introduced a wearable, bidirectional closed‐loop neuromodulation platform called Neuro‐stack, which addressed a major limitation of previous studies by enabling the recording of single‐neuron activity in freely moving human subjects (Figure 6e).^[^ ^213^ ^]^ The Neuro‐stack system comprises 256 electrode contacts, supporting up to 128 monopolar or bipolar recording configurations. Single‐unit activity and LFPs are simultaneously recorded from 32 channels (Figure 6f). Using this device, a range of tasks—including an ambulatory working memory task, closed‐loop neuromodulation, and a stationary verbal memory task—were performed. Neural activity recorded from the medial temporal lobe (MTL) was utilized to predict individual memory performance. In closed‐loop neuromodulation task, stimulation is triggered based on the calculated theta‐band power extracted from the recorded LFP signal. The Neuro‐stack was applied to 12 patients with epilepsy, but only 3 received stimulation, which consisted of 0.5 mA at 100 Hz delivered to the left hippocampus. The proposed BMI system addresses previous technical limitations in recording single‐neuron activity and LFP power in freely moving epilepsy patients. It enables real‐time analysis of these signals and provides a platform for delivering stimulation in a closed‐loop manner. Therefore, it shows strong potential as a next‐generation therapeutic BMI even though only ambulatory working task, in vivo sensing and stimulation, and stationary verbal memory task are demonstrated in the study.

#### 3.3.3 Augmented BMI

Many researchers have attempted to introduce virtual (VR) and augmented reality (AR) to BMI. The application scope of brain–machine interfaces (BMIs) has expanded and now includes use cases such as drone control. However, with the rapid advancement of BMI technologies, conventional silicon chip–based decoders face challenges in processing large‐scale neural data and executing complex decoding algorithms efficiently. Therefore, Liu et al. developed a low‐power, high‐speed 128‐k cell memristor‐based adaptive neuromorphic decoder, and applied it to the BMI for drone control (Figure 6g).^[^ ^214^ ^]^ A total of 64 EEG channels were used to record whole‐head neural signals. Among them, 8 channels were allocated for steady‐state visually evoked potential (SSVEP) detection, while 32 channels were used to capture error‐related potential (ErrP) signals. When a periodic visual stimulus is presented, the visual cortex generates brain signals that are synchronized to the stimulus frequency. These steady‐state visually evoked potentials (SSVEPs) were used as the primary component for decoding drone control commands. Task‐related component analysis (TRCA), a supervised learning algorithm, was employed to extract and decode consistent SSVEP patterns in order to estimate the user's attentional focus. Figure 6h shows the step of template matching for SSVEP signals corresponding to 12 drone control commands. Based on the template, a four‐degrees‐of‐freedom real‐time brain‐controlled drone flight is demonstrated. ErrP is an ERP signal that arises when a command is incorrectly decoded, and it was used to update the decoder accordingly. In addition, Lai et al. investigated the mental simulation mechanism by examining whether the position of an object in a virtual environment could be controlled, based on the understanding that the hippocampus plays a critical role in memory recollection and experiential imagery.^[^ ^215^ ^]^ They recorded spike train data from a rat implanted with a 128‐channel multielectrode array in the CA1 region of the hippocampus while the animal was positioned in specific physical locations. Subsequently, using a deep neural network decoder trained on pre‐recorded spike train data associated with imagined object locations in a virtual space, the rat's location was decoded at 100‐ms intervals. This enabled the successful performance of both the Jumper and Jedi tasks (Figure 6i). These findings demonstrate that objects in a virtual environment can be controlled using neural signals via the BMI system.

## 4 Conclusion

As the healthcare industry undergoes a profound transformation driven by global population aging and the shift toward personalized, precision medicine, bioelectronics is emerging as a pivotal enabler of next‐generation therapeutic solutions. Within this evolving landscape, multi‐channel neural interfaces stand out as transformative tools in neuroscience, enabling the simultaneous recording and modulation of neural activity across multiple regions of the brain and spinal cord. By capturing rich, high‐resolution neural information, these platforms have expanded our ability to probe the spatiotemporal dynamics of neural circuits and to inform the design of targeted, patient‐specific neuromodulation strategies. Recent advances in device engineering—such as flexible substrates, high‐density microelectrode arrays, and the integration of multifunctional capabilities including drug delivery, optical stimulation, and chemical sensing—have further enhanced the performance, versatility, and translational potential of these systems. Coupled with progress in real‐time signal acquisition and AI‐driven computational analysis, multi‐channel neural interfaces are increasingly positioned at the forefront of digital neurotherapeutics and intelligent neuroengineering, paving the way for more adaptive and personalized interventions in the near future.

Despite these promising developments, several critical challenges persist. Long‐term biocompatibility remains a significant concern, particularly for invasive devices, where the mechanical mismatch between rigid materials and soft neural tissue can induce chronic immune responses, ultimately compromising signal fidelity. While emerging soft materials such as polymers, hydrogels, and liquid metals show considerable potential in mitigating these issues, continued refinement is necessary to achieve reliable chronic stability and biological integration. Simultaneously, the vast volume of neural data generated by multi‐channel systems presents substantial hurdles in data analysis and interpretation. Conventional signal processing methodologies are often inadequate for decoding the complex, high‐dimensional interactions within neural networks. Although machine learning and artificial intelligence techniques offer promising avenues for pattern recognition, connectivity mapping, and neural state decoding, their robustness, scalability, and generalizability across subjects and experimental paradigms remain open challenges. Furthermore, as these platforms increasingly support personalized therapeutic applications, the collection, storage, and use of sensitive neural data raise significant ethical concerns regarding privacy, informed consent, and the potential for misuse, underscoring the need for robust governance and data protection frameworks alongside technological advancement.

Looking forward, the advancement of multi‐channel neural interfaces will depend on the seamless integration of hardware, software, and biological systems. Innovations in device miniaturization, wireless data transmission, and low‐power operation will be essential for enabling untethered and real‐world applications. Moreover, the incorporation of adaptive artificial intelligence algorithms capable of online learning and closed‐loop feedback will be critical for realizing responsive neuromodulation strategies that adapt to dynamically changing physiological states. Future strategies are expected to emphasize enhancing chronic stability through bioinspired material designs, increasing electrode density without compromising tissue compatibility, and implementing adaptive closed‐loop control algorithms to enable personalized neuromodulation in real time. Representative development trends also include the integration of multimodal capabilities such as drug delivery, optical stimulation, and chemical sensing within flexible and stretchable platforms, the adoption of self‐healing and viscoplastic materials to prolong device lifespan, and the development of wireless, high‐channel‐count architectures combined with on‐chip processing for autonomous operation. Such developments hold significant promise for enabling personalized therapeutic interventions in a wide range of neurological and psychiatric conditions.

In parallel, the convergence of multi‐channel neural interfaces with BMI technologies is accelerating the evolution of next‐generation neurotechnology. As foundational components of BMI systems, these interfaces offer the rich, multi‐dimensional neural datasets required for the accurate decoding of motor intention, sensory perception, and cognitive states. When integrated with actuators or neural stimulators, they facilitate bidirectional communication between the nervous system and external devices, supporting not only assistive technologies but also restorative interventions.

In conclusion, multi‐channel neural interfaces are poised to reshape the future of both basic neuroscience and clinical neuromodulation. By continuing to address existing limitations through interdisciplinary innovation, these platforms will open new frontiers in understanding brain function, treating disease, and developing intelligent, adaptive neurotechnological systems.

## Conflict of Interest

The authors declare no conflict of interest.
